# Environmental Impacts of Plant-Based Diets: How Does Organic Food Consumption Contribute to Environmental Sustainability?

**DOI:** 10.3389/fnut.2018.00008

**Published:** 2018-02-09

**Authors:** Camille Lacour, Louise Seconda, Benjamin Allès, Serge Hercberg, Brigitte Langevin, Philippe Pointereau, Denis Lairon, Julia Baudry, Emmanuelle Kesse-Guyot

**Affiliations:** ^1^Equipe de Recherche en Epidémiologie Nutritionnelle (EREN), Centre d’Epidémiologie et Statistiques Sorbonne Paris Cité, INSERM (U1153), INRA (U1125), CNAM, Université Paris 13, COMUE Sorbonne Paris Cité, Bobigny, France; ^2^Agence de l’Environnement et de la maîtrise de l’Energie, Angers, France; ^3^Département de Santé Publique, Hôpital Avicenne, Bobigny, France; ^4^Solagro, Toulouse, France; ^5^Nutrition Obésité et Risque Thrombotique (NORT), Aix Marseille Université, INRA 1260, INSERM UMR S 1062, Marseille, France

**Keywords:** provegetarian dietary pattern, organic food consumption, eco-friendly farming, diet-related environmental impact, sustainability

## Abstract

**Background:**

Studies investigating diet-related environmental impacts have rarely considered the production method of the foods consumed. The objective of the present study, based on the NutriNet-Santé cohort, was to investigate the relationship between a provegetarian score and diet-related environmental impacts. We also evaluated potential effect modifications on the association between a provegetarian score and the environmental impacts of organic food consumption.

**Methods:**

Food intake and organic food consumption ratios were obtained from 34,442 French adults using a food frequency questionnaire, which included information on organic food consumption for each group. To characterize the overall structure of the diets, a provegetarian score was used to identify preferences for plant-based products as opposed to animal-based products. Moreover, three environmental indicators were used to assess diet-related environmental impacts: greenhouse gas (GHG) emissions, cumulative energy demand (CED), and land occupation. Environmental impacts were assessed using production life cycle assessment (LCA) at the farm level. Associations between provegetarian score quintiles, the level of organic food consumption, and environmental indicators were analyzed using ANCOVAs adjusted for energy, sex, and age.

**Results:**

Participants with diets rich in plant-based foods (fifth quintile) were more likely to be older urban dwellers, to hold a higher degree in education, and to be characterized by an overall healthier lifestyle and diet. A higher provegetarian score was associated with lower environmental impacts (GHG emissions_Q5vsQ1_ = 838/1,664 kg CO_2eq_/year, −49.6%, *P* < 0.0001; CED_Q5vsQ1_ = 4,853/6,775 MJ/year, −26.9%, *P* < 0.0001; land occupation_Q5vsQ1_ = 2,420/4,138 m^2^/year, −41.5%, *P* < 0.0001). Organic food consumption was also an important modulator of the relationship between provegetarian dietary patterns and environmental impacts but only among participants with diets rich in plant-based products.

**Conclusion:**

Future field studies should endeavor to integrate all the components of a sustainable diet, i.e., both diet composition and production methods.

## Introduction

According to the Food and Agricultural Organization (FAO), adopting sustainable diets at a global level is urgently needed (1). Sustainable diets should include a large share of ecologically based, local and minimally processed products and limited consumption of animal products. Sustainable diets are also healthy in terms of both nutrition and sanitary quality (1). Regarding the environmental aspects of a sustainable diet, a shift from current dietary patterns toward more environmentally friendly habits appears necessary. Environmentally friendly habits include reducing the consumption of animal products and increasing the consumption of plant products (2). Indeed, livestock is considered to be responsible for 18% of the greenhouse gas (GHG) emissions from anthropogenic sources at the global level, and this pattern is comparable at the country level in France (3). More specifically, beef and milk production represent 41 and 20% of the emissions from livestock, respectively (3). Livestock requires substantial energy for multiple activities such as the production of feed, breeding activities, production and spread of fertilizers, electricity use, and operating costs of farm buildings (4). Intensive livestock production is also responsible for a large part of the loss in biodiversity due to important land use for grass and feed crops (5). Conversely, extensive livestock farming is suggested to have positive effects on biodiversity. Studies investigating these issues have consistently reported that decreasing the consumption of animal products would have a considerable beneficial impact on the environment (6, 7).

The FAO also mentions that alternative modes of production may be important to the promotion and development of sustainable diets. Organic agriculture is defined as a system that relies on ecosystem services rather than external agricultural inputs (8). It is generally considered a more environmentally friendly production model that enhances the quality of soil leading to higher plant and fauna diversity and lower nitrate leaching. Nevertheless, disparities in agro-ecological practices still remain (9–12). The sustainability of organic food systems and their ability to feed the global population have often been questioned mostly due to their usually lower crop yields (13, 14). It is now largely recognized that organic production requires less energy inputs than conventional systems (15–17), although benefits in terms of GHG reduction are not straightforward (18). Moreover, firm conclusions about conventional and organic systems are moderated by the functional unit (18–20).

Despite ample literature on environmentally sustainable diets, few studies have considered both dietary patterns and production modes. It is, therefore, of interest to study both parameters simultaneously to be able to estimate the extent to which organic food consumption affects diet-related environmental impacts. It is of considerable interest to consider both plant-based and organic foods, which are consumed by vegetarians in Western countries (21).

A review of Aleksandrowicz et al. revealed that the change from a traditional western diet to alternative dietary patterns (e.g., Mediterranean, vegetarian, or vegan) normally provides benefits for both individual health and the environment (22). The reductions in environmental footprints should generally be proportional to the magnitude of the restriction of animal-based products (22). Despite lower environmental impacts when compared to omnivorous diets (23), vegan or vegetarian diets are still not culturally accepted, particularly in France, where meat-based meals and cheese are an integral part of the traditional diet (24). In this context, the provegetarian score (25), which characterizes diets by the level of plant and animal product consumption, and not directly by animal product exclusion, is highly relevant in the French environment.

Thus, the first objective of this study is to estimate diet-related environmental effects according to the provegetarian score. Second, we focused on studying the moderating effects of organic food consumption according to the level of plant-based food consumption. Data are based on a large sample from the NutriNet-Santé study within the framework of the BioNutriNet project, which enabled us to collect food consumption data and environmental data on both organic and conventional products.

## Materials and Methods

### Study Population

The subjects are adult volunteer participants from the prospective NutriNet-Santé cohort, which was launched in May 2009 in France. The NutriNet*-*Santé study has been previously described in detail in another study (26). At inclusion in the cohort and yearly thereafter, the participants completed three 24-h randomly distributed accounts that were provided over a period of 15 days. They were also asked to complete a set of questionnaires about their sociodemographics, anthropometrics, health status, and lifestyle characteristics. Participants were also regularly invited to complete complementary questionnaires. In 2014, participants were asked to provide information on their organic food consumption as well as their motives and attitudes toward organic foods.

### Data Collection

#### Sociodemographic and Lifestyle Characteristics

The yearly updated inclusion questionnaire provided data on sociodemographic characteristics including age, sex, highest achieved degree (lower than high school, high school, or post-secondary graduate), location (rural community, urban unit with a population smaller than 20,000 inhabitants, between 20,000 and 200,000 inhabitants, or higher than 200,000 inhabitants), and monthly income per household unit (lower than 900 euros, between 900 and 1,200 euros, between 1,200 and 1,800 euros, between 1,800 and 2,700 euros, and higher than 2,700 euros). The monthly income per household unit was obtained by dividing monthly income by consumption units (CU); the first adult in the household represents 1 CU, other persons older than 14 represent 0.5 CU, and other persons younger than 14 years represent 0.3 CU (27).

This set of data also provided lifestyle characteristics such as physical activity (measured by the IPAQ–International Physical Activity Questionnaire) (28–30), smoking status (never, former, and current smoker), and alcohol intake (never, moderate, or frequent drinker). Moderate alcohol consumption was defined as an intake lower than 20 g/day for women and lower than 30 g/day for men (31).

#### Dietary Data and Organic Food Consumption

Between June and December 2014, participants were asked to complete an optional organic food semi-quantitative frequency questionnaire (Org-FFQ) based on the original validated Nutrinet FFQ (32). The Org-FFQ collected information on consumption frequencies (yearly, monthly, weekly, and daily units) and portion sizes for 264 food and beverage items over a year. The total food intake was estimated by multiplying the consumption frequency and portion size for each item. To estimate the share of organic food consumption in the diet, for each item in the Org-FFQ, participants indicated how often they consumed that item in an organic form. Organic food frequency was assessed using a 5-point ordinal scale, “never,” “rarely,” “half of the time,” “often,” and “always,” which were weighted as 0, 0.25, 0.5, 0.75, and 1, respectively, and yielded an estimate of the proportion of organic food consumed in an individual diet. The contribution of organic food consumption to the diet was calculated by dividing the total organic food intake (g/day) by the total food intake (g/day) excluding water. This ratio was multiplied by 100 to obtain the contribution of organic food as a percentage of weight.

The development of the Org-FFQ and sensitivity analyses for the allocation of arbitrary weightings has been described in another study (21).

The NutriNet-Santé food composition database (33) was used to estimate daily nutrient intake independently of the production method. To assess the nutritional quality of dietary patterns, two indicators were assessed at the individual level: the PANDiet (based on the probability of adequate nutrient intake for 24 nutrients) (34) and the mPNNS-GS (modified French national nutrition and health programme (Programme National Nutrition Santé), with the PNNS-guidelines score based on the adherence to the PNNS recommendations excluding physical activity) (35).

#### Environmental Data

The methodology for the environmental evaluation of individual diets is described in detail in the Presentation S1 in Supplementary Material. Data were collected *via* the French diagnostic tool DIALECTE (36) using the life cycle assessment method (LCA) (37, 38) at the farm level (from agricultural inputs and animal feed production to harvest). To date, DIALECTE is the only French database that covers such a large panel of data for both organic and conventional agricultural products. This study considers the three environmental indicators available: (1) GHG emissions were estimated including carbon dioxide, methane and nitrous oxide emissions and were expressed in kilogram CO_2_ equivalent per day. (2) The cumulative energy demand (CED) indicator was defined as the consumption of renewable and unrenewable energy in megajoules per day according to the CED method (39). (3) Finally, land occupation was defined as the area in square meters needed per day. The environmental database includes information on 62 raw agricultural products based on measurements from 2,086 farms in France and on 30 raw agricultural products based on information from the literature. Among these farms, 46% follow certified organic agricultural practices (as defined by European regulations).

For each participant, organic and conventional food consumption was multiplied by the environmental impact of each product to estimate the impact of the overall diet for each participant.

### Construction of the Provegetarian Score

The provegetarian score was developed to reflect the proportion of plant-based food consumed in a diet (25). Components of the provegetarian score include seven vegetable food groups and five animal food groups (25) (Table S1 in Supplementary Material). Sex-specific adjustment for total energy intake was made for the consumption of each food group using the residual method (40). Energy-adjusted, sex-specific quintile values for each plant component were calculated by allocating 1 to 5 points. For animal food groups, the quintile values were reversed (from 5 for the first quintile to 1 for the fifth quintile). Finally, the provegetarian score was obtained by summing the quintile value of each vegetable food group and the reverse quintile value of each animal food group. The score ranges from 12 (low consumption of plant food) to 60 (high consumption of plant food).

### Data Treatment and Statistical Analysis

Among the 37,685 participants who completed the Org-FFQ, participants with missing sociodemographic data or aberrant data were excluded (*N* = 1,390). To detect under reporting and over reporting, energy requirements were calculated for each individual using physical activity level (IPAQ) and basal metabolic rate, estimated by Schofield’s equation (41) and taking into account age, sex, and BMI. The ratio of energy intake to energy requirement was calculated, and participants with a ratio below 0.35 or above 1.93 were excluded (*N* = 1,099). Finally, only participants living in mainland France and having complete data to calculate the nutritional quality scores were included. The final sample included 34,442 participants, with 22,813 women and 7,569 men.

Sociodemographic and lifestyle characteristics along with food and nutrient intakes were presented across the provegetarian score quintiles. For descriptive purposes, nutrient and food data were adjusted for total energy intake by sex using the residual method (40). Means, SDs and percentages were provided as appropriate. *P* values referred to the Mantel–Haenzel chi-square trend test for categorical variables and to the linear contrast test (ANCOVA) for continuous variables.

The contributions (as percentages) of different food groups to diet-related GHG emissions and CED across provegetarian score quintiles are presented. All *P*-trends were obtained with linear contrast tests (ANCOVA).

As an interaction between the provegetarian score and organic food consumption was observed (*P* ≤ 0.0001), data were stratified by the level of organic food consumption. Associations between the provegetarian score and environmental impacts for the overall sample and the stratified tertiles of the contribution of organic consumption to the whole diet were estimated using ANCOVA adjusted with Dunett’s test. All models were adjusted for sex, age, and energy intake. In addition, the ratio of organic food consumption as a continuous variable was included in the stratified analyses to account for residual confounding. The ordinal margins option was used. In all the analyses, the environmental indicators were log-transformed to improve the normality of the distributions. The data are presented as adjusted means with their 95% confidence intervals. Unadjusted models are provided in the Table S2 in Supplementary Material. Two-sided tests and a *P-*value <0.05 were used for statistical significance.

All analyses were performed using SAS software (SAS Institute Inc., Cary, NC, USA).

## Results

### Individual Characteristics

Table 1 presents sociodemographic and lifestyle characteristics of the participants across provegetarian score quintiles. No difference in the sex distribution of participants across the quintiles was observed. Participants with higher provegetarian scores were more likely to be highly educated, physically active, non-smokers, and moderate or non-drinkers. The Q5 of the provegetarian score (reflecting high consumption of plant food) included the highest proportion of participants with the lowest monthly income per household unit (<900 euros), while Q4 included the highest proportion of participants with the highest monthly income (>2,700 euros). The highest proportion of participants living in population-dense urban units was found in the Q4. Finally, the largest share of vegetarians was included in the Q5 of the provegetarian diet (8.3% in Q5 versus 0.2% in Q1).

**Table 1:** Sociodemographic and lifestyle characteristics by provegetarian score quintile, *N* = 34,442, BioNutriNet study, 2014.^a^

	Q1	Q2	Q3	Q4	Q5	*P*-trend^b^
*N* (%)	(17.8)	(23.6)	(20.2)	(16.8)	(21.6)	
**Provegetarian score**						
Mean	27.4 (2.5)	32.6 (1.1)	36.0 (0.8)	38.9 (0.8)	44.5 (3.5)	<0.0001
Median (IQR)	28 (3)	33 (2)	36 (2)	39 (2)	44 (2)	
**Sex (%)**						
Female	75.8	75.5	75.2	75.3	76.0	0.81
Male	24.2	24.5	24.8	24.7	24.0	
**Age (years)**	52.0 (14.0)	53.0 (14.1)	53.5 (14.0)	54.5 (13.6)	53.4 (14.1)	<0.0001
**Education level (%)**						
<High-school diploma	21.6	21.5	21.5	21.0	19.1	<0.0001
High-school diploma	15.9	15.4	14.3	13.8	14.1	
Post-secondary graduate	62.5	63.1	64.1	65.2	66.8	
**Monthly income per household unit (%)**						
Refuse to declare	5.8	6.2	6.3	5.7	7.1	0.01
<900 euros	7.4	6.6	6.0	6.3	8.7	
900–1,200 euros	5.2	4.4	4.6	4.4	4.7	
1,200–1,800 euros	24.2	23.1	23.6	22.1	22.4	
1,800–2,700 euros	27.3	27.7	26.7	27.7	26.9	
>2,700 euros	30.1	31.9	32.9	33.7	30.3	
**Location (%)**					
Rural community	22.9	23.2	22.3	20.7	22.3	0.11
Urban unit with a population of <20,000 inhabitants	16.1	15.4	15.7	15.0	15.2	
Urban unit with a population of 20,000–200,000 inhabitants	17.8	18.5	18.4	18.8	19.0	
Urban unit with a population of >200,000 inhabitants	43.2	42.9	43.6	45.6	43.5	
**Physical activity (%)**						
Missing value	11.6	11.5	10.9	10.3	9.9	<0.0001
Low	22.9	21.7	19.5	17.4	15.2	
Medium	35.3	36.3	37.2	37.2	37.2	
High	30.2	30.5	32.4	35.1	37.6
**Smoking status (%)**						
Non-smoker	48.6	48.5	48.4	48.6	49.3	0.04
Former smoker	13.3	11.6	11.2	9.7	9.0	
Smoker	38.0	39.8	40.5	41.7	41.7	
**Alcohol intake (%)**					
Non-drinker	4.9	4.8	5.2	4.6	7.9	<0.0001
Moderate drinker (<20 g/day for women and <30 g/day for men)	83.5	86.0	86.1	88.0	85.8	
High drinker (>20 g/day for women and >30 g/day for men)	11.6	9.2	8.7	7.4	6.4
**Diet (%)**					
Vegetarians	0.18	0.39	0.83	1.46	8.27	<0.0001
Vegans	0.00	0.01	0.04	0.12	5.28

*IQR, interquartile range; Q, quintile of provegetarian score*.

*^a^Values are presented as the mean (SD) or as a percentage*.

*^b^Values based on linear contrast test or γ^2^*.

### Food and Nutrients Intake by Provegetarian Score Quintile

Tables 2 and 3 present food groups and nutrient intake across provegetarian score quintiles. By construction, the consumption of animal-based products decreased while the consumption of plant-based products increased across quintiles. Participants in the highest quintile also consumed less fast food products (hamburgers, pizzas, and sandwiches), sweets, and alcohol and had a higher intake of salad dressings, popcorn, or nuts. Overall, considering nutrient intake, a higher provegetarian score was associated with a lower overall protein intake but a higher proportion of plant protein (50.5% in Q5 versus 22.2% in Q1) and a higher polyunsaturated fatty acid (PUFA) and monounsaturated fatty acid (MUFA) intake as well as a lower saturated fatty acid intake and higher n-6/n-3 PUFA ratio. The intake of carbohydrates and fiber increased across provegetarian score quintiles. Participants in the Q5 of the provegetarian score also displayed the highest level of organic food consumption, as organic food represented a 50% share of their total food consumption.

**Table 2:** Food and nutrient intake by provegetarian score quintile, *N* = 34,442, BioNutriNet study, 2014.^a^

	Q1	Q2	Q3	Q4	Q5	*P*-trend^b^
**Nutrients intake**						
Energy intake without alcohol (kcal/day)	2,218.8 (638.5)	1,969.9 (597.1)	1,885.6 (614.0)	1,868.2 (599.6)	1,982.9 (613.6)	<0.0001
Proteins (%)^c^	20.7 (3.6)	19.5 (3.3)	18.5 (3.2)	17.5 (2.9)	15.6 (2.9)	<0.0001
Plant proteins (% protein)^c^	22.2 (6.3)	27.0 (7.3)	31.3 (8.3)	36.1 (9.3)	50.5 (17.9)	<0.0001
Animal proteins (% protein)^c^	77.8 (6.3)	73.0 (7.3)	68.8 (8.3)	63.9 (9.3)	49.5 (17.9)	<0.0001
Lipids (%)^c^	41.2 (6.4)	40.2 (6.7)	39.8 (7.1)	39.9 (7.2)	40.2 (7.5)	<0.0001
Plant lipid (% of lipid)^c^	34.4 (10.4)	40.7 (10.8)	46.1 (11.5)	51.2 (12.0)	63.3 (15.0)	<0.0001
Animal lipid (% of lipid)^c^	65.6 (10.4)	59.3 (10.8)	53.9 (11.5)	48.8 (12.0)	36.7 (15.0)	<0.0001
PUFA (%)^c^	5.6 (1.7)	6.1 (2.0)	6.4 (2.3)	6.9 (2.4)	8.3 (3.0)	<0.0001
MUFA (%)^c^	15.4 (3.2)	15.5 (3.6)	15.8 (4.0)	16.2 (4.2)	17.1 (4.6)	<0.0001
SFA (%)^c^	17.0 (3.5)	15.6 (3.1)	14.6 (3.1)	13.8 (3.0)	12.0 (3.1)	<0.0001
Omega 3 (%)^c^	2.0 (0.9)	2.2 (1.0)	2.3 (1.1)	2.5 (1.2)	3.0 (1.6)	<0.0001
Omega 6 (%)^c^	10.9 (3.0)	12.1 (3.5)	13.0 (3.9)	14.0 (4.0)	16.8 (5.1)	<0.0001
Ratio n-6/n-3	6.3 (2.7)	6.5 (3.0)	6.6 (3.1)	6.5 (3.1)	6.7 (3.3)	<0.0001
Carbohydrates (%)^c^	35.3 (7.1)	37.5 (7.2)	39.0 (7.2)	40.0 (7.3)	41.9 (7.6)	<0.0001
Fibers (%)^c^	1.7 (0.5)	2.0 (0.6)	2.2 (0.6)	2.5 (0.7)	3.0 (0.8)	<0.0001
Alcohol (g/day)	9.9 (14.8)	8.9 (12.6)	8.5 (13.2)	7.95 (10.7)	7.17 (10.3)	<0.0001
**Food consumption (g or ml/day)**^d^						
Vegetables and fruits	424.4 (301.7)	564.3 (314.9)	660.6 (346.0)	734.8 (354.1)	881.7 (420.0)	<0.0001
Meat	165.3 (85.4)	139.4 (65.8)	123.3 (60.2)	109.2 (54.2)	71.9 (54.8)	<0.0001
Ruminant (%)	36.0 (15.7)	35.4 (16.0)	35.3 (15.9)	34.2 (16.4)	32.1 (18.1)	<0.0001
Pork (%)	42.1 (15.9)	41.8 (16.2)	41.1 (16.6)	41.4 (17.4)	40.8 (19.9)	<0.0001
Poultry (%)	20.7 (13.9)	21.4 (14.5)	22.1 (15.5)	22.9 (16.1)	25.5 (19.3)	<0.0001
Rabbit (%)	1.2 (2.4)	1.4 (2.7)	1.5 (2.7)	1.5 (2.6)	1.6 (3.7)	<0.0001
Eggs	13.5 (15.2)	11.2 (11.7)	10.1 (10.8)	9.4 (9.8)	8.4 (11.0)	<0.0001
Fish	53.6 (56.3)	49.3 (39.1)	47.1 (39.8)	45.4 (34.3)	37.4 (38.0)	<0.0001
Dairy products	320.6 (248.1)	265.7 (209.3)	227.6 (184.4)	185.5 (161.0)	112.1 (145.0)	<0.0001
Starchy food	159.9 (82.4)	179.1 (86.7)	189.8 (89.1)	194.6 (91.4)	213.1 (110.6)	<0.0001
Whole cereal products	33.3 (54.2)	47.0 (62.0)	54.0 (62.4)	63.7 (68.0)	84.1 (81.7)	<0.0001
Soy products	1.1 (54.2)	8.5 (61.7)	16.2 (76.8)	27.2 (97.7)	70.6 (140.7)	<0.0001
Fast food	38.7 (48.1)	36.1 (31.6)	33.9 (33.1)	32.7 (29.0)	27.3 (24.2)	<0.0001
Nuts	2.00 (8.63)	4.03 (8.93)	5.39 (10.79)	7.14 (13.19)	11.36 (15.25)	<0.0001
Extra food (excluding nuts)	9.55 (9.26)	9.84 (9.80)	9.93 (9.51)	9.59 (9.99)	8.50 (9.07)	<0.0001
Sweet products	80.6 (60.6)	77.3 (52.6)	74.0 (46.3)	69.7 (41.2)	61.9 (39.1)	<0.0001
Oil	8.9 (12.2)	13.0 (12.5)	15.8 (13.3)	18.3 (14.1)	22.7 (15.5)	<0.0001
Butter	8.6 (7.9)	7.4 (6.7)	6.7 (6.2)	6.2 (6.1)	4.5 (5.6)	<0.0001
Other fats	2.4 (4.6)	2.3 (4.7)	2.4 (4.8)	2.1 (3.9)	2.1 (4.6)	0.1
Non-alcoholic drink	1571 (769)	1600 (763)	1590 (739)	1607 (731)	1591 (755)	0.1
Alcoholic drink	180.7 (162.6)	177.7 (142.6)	174.0 (144.4)	170.5 (118.5)	158.1 (114.8)	<0.0001
**Level of organic food consumption (in % of weight)**	18.2 (19.8)	22.5 (22.2)	26.3 (24.2)	32.1 (26.4)	48.2 (30.7)	<0.0001
Median of organic food consumption	12	17	21	26	48	
IQR	26	32	36	41	53	
**mPNNS score (/13.5)**	7.6 (1.87)	8.3 (1.73)	8.6 (1.67)	8.8 (1.66)	8.8 (1.68)	<0.0001

*PUFA, polyunsaturated fatty acid; MUFA, monounsaturated fatty acid; SFA, saturated fatty acid; IQR, interquartile range; Q, quintile of provegetarian score*.

*^a^Values are presented as the mean (SD)*.

*^b^Values based on a linear contrast test*.

*^c^As percent of energy intake*.

*^d^Values adjusted on the energy intake*.

**Table 3:** Consumption of micronutrients by provegetarian score quintile, *N* = 34,442, BioNutriNet study, 2014.^a^

	Q1	Q2	Q3	Q4	Q5	*P*-trend^b^
**Vitamins**^c^						
Retinol (μg/day)	769.42 (1,530.30)	667.23 (1,052.50)	590.84 (420.54)	558.82 (362.70)	432.86 (353.49)	<0.0001
β-carotene (μg/day)	2,685.90 (2,025.9)	3,579.01 (3,040.9)	4,175.05 (2,459.7)	4,677.09 (2,956.2)	5,872.50 (3,443.6)	<0.0001
Vitamin B1 (mg/day)	1.33 (0.40)	1.35 (0.40)	1.35 (0.38)	1.37 (0.38)	1.48 (0.52)	<0.0001
Vitamin B2 (mg/day)	2.47 (0.70)	2.31 (0.61)	2.20 (0.55)	2.13 (0.52)	1.99 (0.51)	<0.0001
Vitamin B3 (mg/day)	26.61 (8.44)	26.21 (7.29)	25.70 (6.68)	25.64 (6.41)	24.23 (6.30)	<0.0001
Vitamin B5 (mg/day)	6.76 (1.65)	6.54 (1.44)	6.39 (1.33)	6.26 (1.23)	6.06 (1.19)	<0.0001
Vitamin B6 (mg/day)	1.91 (0.50)	1.96 (0.47)	1.99 (0.46)	2.04 (0.46)	2.17 (0.56)	<0.0001
Vitamin B9 (μg/day)	317.56 (122.44)	359.11 (123.69)	385.77 (116.52)	410.20 (123.12)	482.06 (157.47)	<0.0001
Vitamin B12 (μg/day)	8.69 (9.07)	7.67 (6.41)	6.96 (3.14)	6.57 (2.77)	5.23 (2.93)	<0.0001
Vitamin C (mg/day)	104.49 (70.41)	126.24 (70.54)	141.66 (81.21)	151.66 (78.85)	174.60 (90.83)	<0.0001
Vitamin D (μg/day)	3.55 (2.22)	3.24 (1.63)	3.08 (1.70)	2.97 (1.47)	2.54 (1.63)	<0.0001
Vitamin E (mg/day)	9.66 (4.36)	11.59 (4.37)	12.74 (4.63)	13.76 (4.63)	16.44 (5.67)	<0.0001
Vitamin K (μg/day)	142.59 (122.15)	187.11 (143.88)	217.57 (140.31)	244.35 (205.46)	310.09 (201.96)	<0.0001
**Minerals**^c^						
Ca (mg/day)	1,172 (391)	1,094 (329)	1,044 (304)	998 (282)	915 (261)	<0.0001
Fe (mg/day)	14.23 (3.78)	14.92 (3.44)	15.25 (3.25)	15.83 (3.40)	17.36 (4.00)	<0.0001
Haem Fe (mg/day)	1.97 (1.62)	1.68 (0.85)	1.50 (0.75)	1.36 (0.67)	0.94 (0.65)	<0.0001
I (μg/day)	160.20 (280.37)	177.08 (211.47)	193.07 (275.48)	203.11 (309.02)	329.66 (710.17)	<0.0001
Mg (mg/day)	444.43 (138.26)	470.93 (134.19)	482.83 (130.29)	502.58 (132.78)	540.85 (140.23)	<0.0001
P (mg/day)	1,550.19 (315.86)	1,471.20 (270.59)	1,420.12 (255.63)	1,379.49 (238.06)	1,322.24 (234.55)	<0.0001
K (mg/day)	3,508.30 (840.07)	3,645.63 (825.93)	3,726.91 (831.23)	3,802.52 (835.48)	3,961.31 (904.80)	<0.0001
Na (mg/day)	2,739.20 (592.86)	2,641.56 (492.86)	2,570.26 (491.03)	2,515.43 (475.96)	2,290.61 (550.78)	<0.0001
Cu (mg/day)	1.74 (1.48)	1.90 (1.09)	1.98 (0.58)	2.10 (0.54)	2.38 (0.62)	<0.0001
Zn (mg/day)	13.98 (3.13)	13.30 (2.60)	12.81 (2.31)	12.52 (2.19)	11.85 (2.23)	<0.0001
Mn (mg/day)	3.65 (1.93)	4.40 (1.95)	4.84 (1.91)	5.32 (2.01)	6.53 (2.47)	<0.0001
Se (μg/day)	83.19 (26.28)	80.68 (20.30)	78.94 (20.02)	78.04 (18.69)	75.43 (19.51)	<0.0001
**PANDiet score (/100)**	62.43 (5.13)	64.90 (5.99)	66.37 (6.72)	67.99 (7.07)	71.12 (7.13)	<0.0001

*^a^Values are presented as the mean (SD)*.

*^b^Values based on a linear contrast test*.

*^c^Energy-adjusted mean (SD)*.

Considering vitamins and minerals, iron intake increased across the quintiles of the provegetarian score while haem iron decreased. As expected, participants in the Q5 of the provegetarian score also exhibited a higher intake of most micronutrients (β-carotene, B1, B6, B9, C, E, K vitamins, and minerals Mg, K, and Mn). According to both the mPNNS-GS and PANDiet scores, participants in the last quintile showed the highest adherence to the French dietary guidelines.

### Environmental Impacts by Provegetarian Score Quintile

After the adjustment for energy intake, age, and sex, diet-related GHG emissions, CED, and land occupation decreased across the provegetarian score quintiles by −49.6, −26.9, and −41.5%, respectively, between Q5 and Q1 (Table 4). For all indicators, a linear association was observed (*P* < 0.0001). This reflects that the richer a diet is in plant products, the lower the environmental impacts are. For informational purposes, crude means and SDs of environmental indicators across the quintiles of the provegetarian score are presented in Figure S1 in Supplementary Material.

**Table 4:** Association between provegetarian score tertile and environmental impacts according to the level of organic food consumption, BioNutriNet study, 2014.

	Overall		Level of contribution of organic food to the diet						
			Low (0.03)		Medium (0.23)		High (0.63)		
	Mean^a^	95% CL	Mean^a^	95% CL	Mean^a^	95% CL	Mean^a^	95% CL	
**Greenhouse gas emissions (CO2eq/day)**									
Q1 provegetarian score	4.56	(4.51–4.60)	4.59	(4.53–4.65)	4.56	(4.48–4.63)	4.10	(3.99–4.22)	
Q2 provegetarian score	4.05	(4.01–4.08)	4.13	(4.08–4.18)	4.05	(4.00–4.10)	3.74	(3.66–3.81)	
Q3 provegetarian score	3.62	(3.62–3.66)	3.73	(3.68–3.78)	3.68	(3.63–3.74)	3.34	(3.28–3.41)	
Q4 provegetarian score	3.23	(3.20–3.27)	3.45	(3.39–3.51)	3.38	(3.33–3.43)	2.94	(2.89–2.99)	
Q5 provegetarian score	2.27	(1.33–2.29)	2.93	(2.87–2.99)	2.72	(2.67–2.76)	2.12	(2.09–2.14)	
P^b^ interaction									<0.0001
P^c^ Q1 vs Q2									0.9711
P^c^ Q1 vs Q3									0.2764
P^c^ Q1 vs Q4									<0.0001
P^c^ Q1 vs Q5									<0.0001
**Cumulative energy demand (MJ/day)**	
Q1 provegetarian score	18.55	(18.43–18.67)	18.58	(18.40–18.75)	18.58	(18.39–18.78)	17.33	(17.05–17.63)	
Q2 provegetarian score	17.43	(17.33–17.53)	17.62	(17.47–17.77)	17.47	(17.32–17.63)	16.53	(16.32–16.73)	
Q3 provegetarian score	16.48	(15.52–16.58)	16.87	(16.70–17.04)	16.62	(16.47–16.78)	15.59	(15.41–15.77)	
Q4 provegetarian score	15.62	(15.52–15.73)	16.42	(16.21–16.63)	16.10	(15.93–16.27)	14.62	(14.45–14.78)	
Q5 provegetarian score	13.29	(13.21–13.37)	15.56	(15.33–15.79)	14.72	(14.56–14.89)	12.66	(12.56–12.76)	
P^b^ interaction									<0.0001
P^c^ Q1 vs Q2									0.9417
P^c^ Q1 vs Q3									0.1044
P^c^ Q1 vs Q4									<0.0001
P^c^ Q1 vs Q5									<0.0001
**Land occupation (m^2^/day)**	
Q1 provegetarian score	11.33	(11.14–11.41)	10.94	(10.78–11.10)	11.58	(11.39–11.78)	11.66	(11.36–11.96)	
Q2 provegetarian score	10.26	(10.17–10.35)	9.89	(9.76–10.03)	10.31	(10.17–10.45)	10.64	(10.45–10.85)	
Q3 provegetarian score	9.34	(9.26–9.43)	8.95	(8.81–9.09)	9.43	(9.29–9.57)	9.61	(9.44–9.79)	
Q4 provegetarian score	8.51	(8.42–8.60)	8.26	(8.10–8.43)	8.68	(8.54–8.83)	8.50	(8.35–8.65)	
Q5 provegetarian score	6.63	(6.57–6.69)	7.03	(6.87–7.19)	7.09	(6.97–7.21)	6.49	(6.41–6.57)	
P^b^ interaction									<0.0001
P^c^ Q1 vs Q2									0.7782
P^c^ Q1 vs Q3									0.9696
P^c^ Q1 vs Q4									0.0111
P^c^ Q1 vs Q5									<0.0001

*Models are adjusted on sex, age, and energy intake*.

*^a^Adjusted means were obtained with ANOVA models by the level of organic food contribution in the diet. P-trends across the provegetarian score quintile are all <0.0001 and were obtained with a linear contrast test by the level of organic food contribution in the diet*.

*^b^P for interaction between provegetarian score quintiles and the level contribution of organic food to the diet*.

^c^P-linear trend of Q*v.Q1 of provegetarian score. It reflects the linearity of the difference between the first and the other quintiles of the provegetarian score across the levels of organic consumption.

### Contribution of Food Groups to Diet-Related GHG Emissions, CED, and Land Occupation by Provegetarian Score Quintile

**Figure 1:**
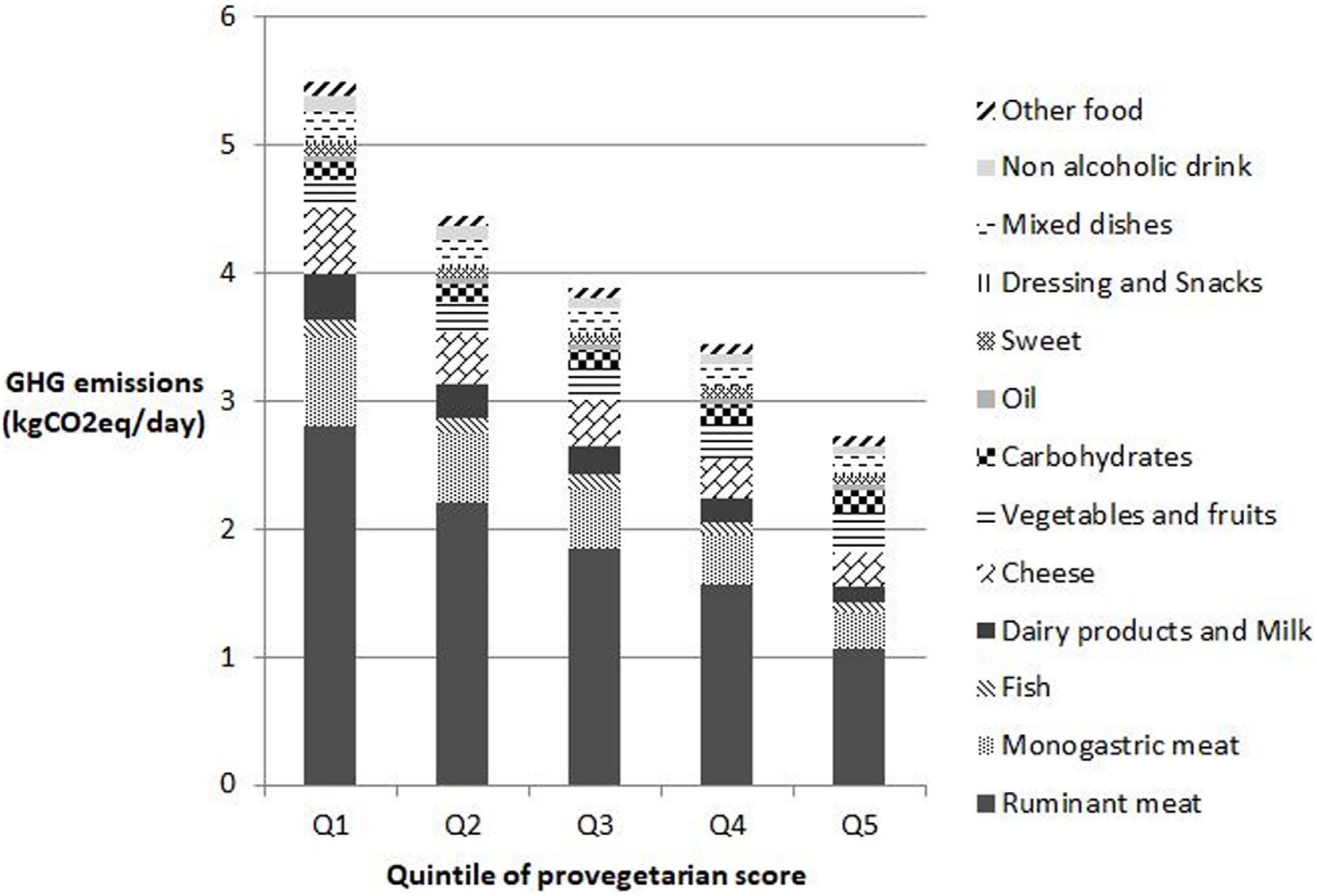
Greenhouse gas (GHG) emissions by food group and by quintile of provegetarian score. Other food group includes whole products, soy products, eggs, butter, other fats, and alcohol. Food group impacts are all significant (*P*-trend < 0.05).

Figure 1 indicates that the main contributor to diet-related GHG emissions across the different provegetarian score categories was animal-based products, particularly ruminant meat consumption. Animal products were responsible for approximately 80% of the dietary GHG emissions for diets rich in animal products (Q1 of the provegetarian score), between 70 and 80% for diets moderate in animal products and approximately 60% for diets rich in plant products (Q5 of the provegetarian score). Specifically, ruminant meat represented approximately half of the diet-related GHG emissions, regardless of the type of diet considered.

**Figure 2:**
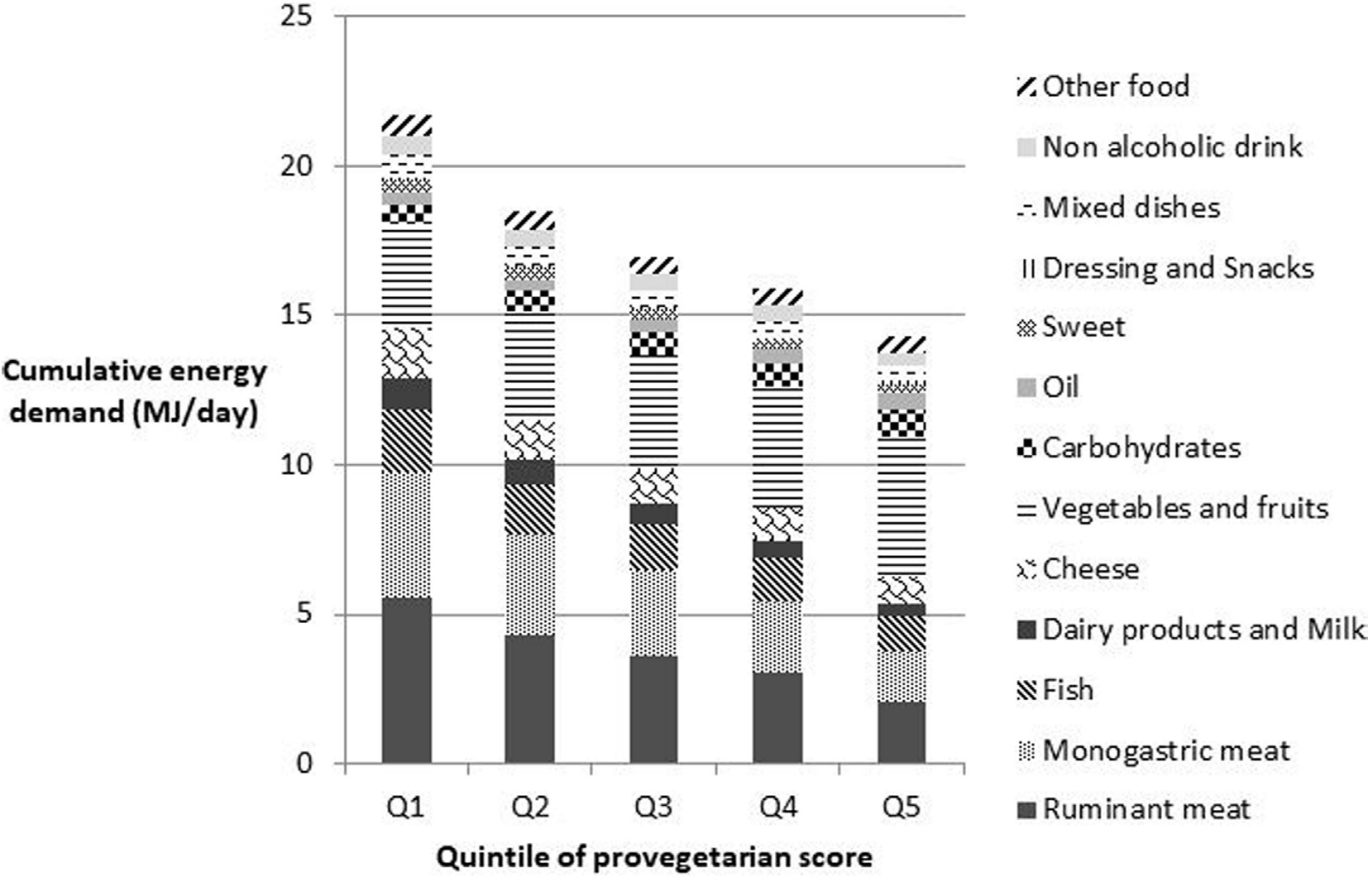
Cumulative energy demand by food group and by quintile of provegetarian score. Other food group includes whole products, soy products, eggs, butter, other fats, and alcohol. Food group impacts are all significant (*P*-trend < 0.05).

Concerning the CED (Figure 2), consumption of fruits, and vegetables was the major contributor (except for Q1 and Q2). Estimates of the contribution of monogastric meat and ruminant meat to diet-related CED were similar.

**Figure 3:**
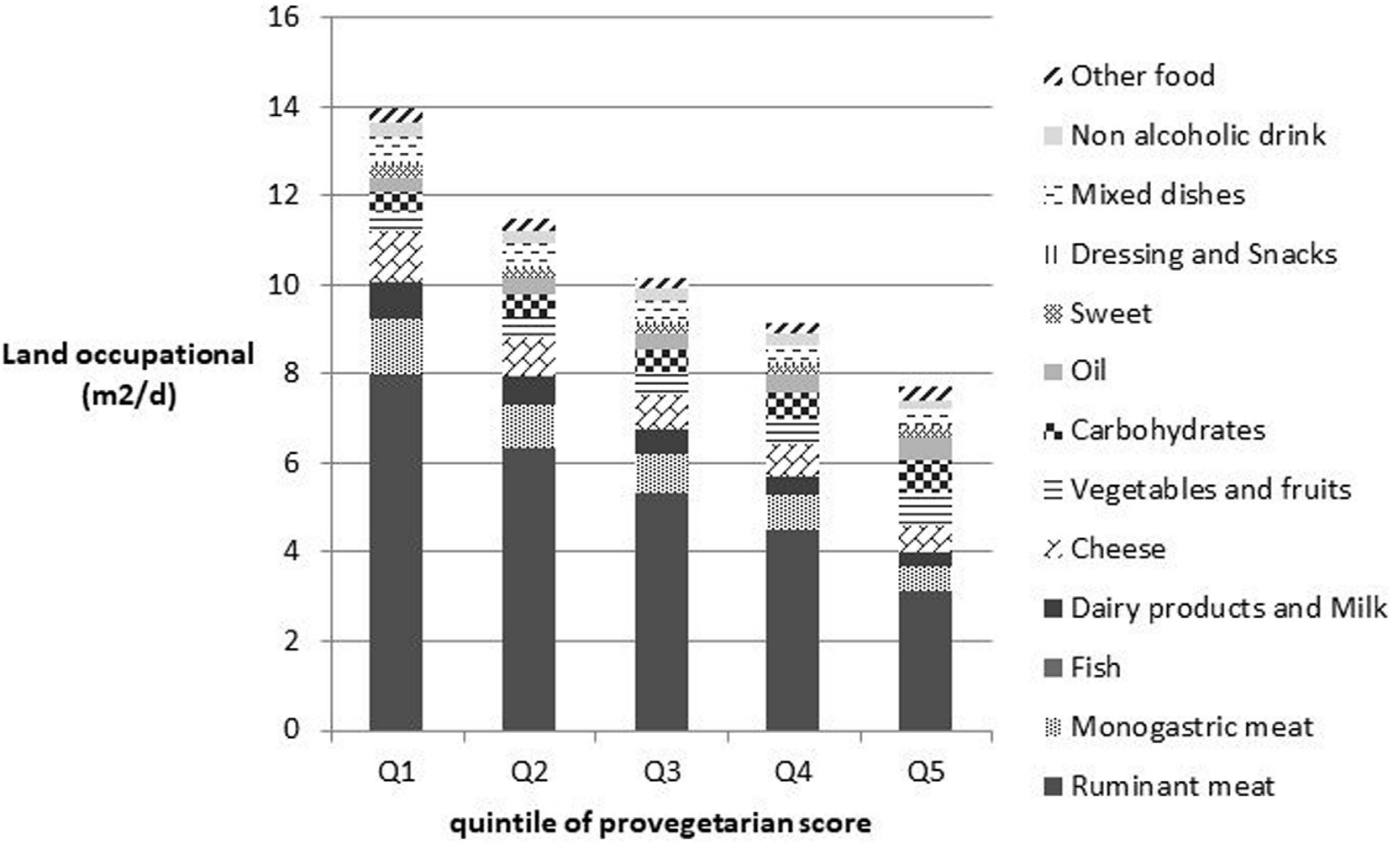
Land occupation by food group and by quintile of provegetarian score. Other food group includes whole products, soy products, eggs, butter, other fats, and alcohol. Food group impacts are all significant (*P*-trend < 0.05).

Finally, Figure 3 presents land occupation by food group and by quintile. The results were closer than those for GHG emissions, showing a high contribution of animal products specifically ruminant meat to land occupation.

Moreover, the contribution of carbohydrates and oil to GHG emissions and CED increased across the provegetarian score quintiles. Of note, the contribution from cheese consumption was more important than from the contribution from dairy products and milk across quintiles.

### Diet-Related Environmental Impacts Considering both the Provegetarian Score and the Level of Organic Food Consumption

Table 4 presents the association between the provegetarian score and the environmental impacts stratified by the level of organic food consumption. Similar linear trends were observed between the provegetarian score and environmental impacts across the different levels of organic food consumption. Considering diet-related environmental impacts in diets that contained low or moderate (Q1, Q2, and Q3) amounts of plant products (i.e., ≥70% animal protein for protein intake and ≥45% animal lipid for lipid intake), the level of organic food consumption did not significantly modify the association between the provegetarian score and the environmental impacts (GHG: P_Q2vsQ1_ = 0.97 and P_Q3vsQ1_ = 0.28; CED: P_Q2vsQ1_ = 0.94 and P_Q3vsQ1_ = 0.10; land occupation P_Q2vsQ1_ = 0.78 and P_Q3vsQ1_ = 0.97). However, for diets rich in plant foods (Q4 and Q5), the differences in the environmental impacts across the provegetarian score quintiles increased across the organic food ratio tertiles (*P* < 0.0001 except for land occupation P_Q4vsQ1_ = 0.01).

## Discussion

In our study, participants with a high provegetarian score were characterized by an overall healthier lifestyle, including healthier diets, as reflected by higher PANDiet and mPNNS-GS scores. Diets rich in plant products displayed lower environmental impacts (GHG emissions, CED and land occupation). Moreover, the consumption level of organic products was shown to have a positive moderating effect on diet-related environmental impacts only in diets rich in plant-based food.

Overall, a higher provegetarian score was associated with lower environmental impacts, particularly GHG emissions, across all levels of organic food consumption. These results at the individual diet level were expected since livestock is the most substantial agricultural contributor to GHG emissions, demands high energy inputs, and requires important land resources (42–44).

Similar results for GHG emissions were documented in the EPIC-Oxford observational study. However, the estimations were not adjusted for energy intake, and the LCA did not consider the production mode even though it included all stages of production, transformation, and distribution. The authors showed that a diet rich in animal products emitted 2.5 times as much GHG than a vegan diet. For women and men, GHG emissions from the diets of meat-eaters were 46 and 51% higher, respectively, than those of fish-eaters (or pesco-vegetarians), and 50 and 54% higher, respectively, than those of vegetarians (45). Other studies documented similar trends in regards to environmental impacts of modeled substitutions for meat (46–48). For instance, the modeled substitution in the EPIC-Netherlands cohort demonstrated that substituting meat with 35 g/d of different combinations of plant products including potatoes, pasta, vegetables, nuts, and milky desserts could reduce GHG emissions up to 12% (49). In a recent review, authors concluded that the isocaloric substitution of meat by starchy food, fruits, nuts, and vegetables was more sustainable in terms of GHG emissions. However, in that same review, production modes (more or less agro-ecological modes) were not distinguished (43).

Livestock results in GHG emissions such as nitrous oxide, carbon dioxide, and methane due to high-energy feed production, concentrating production and enteric fermentation of ruminants (3). However, impacts related to ruminant meat are higher when compared to monogastric animals because of methane emissions and the need for substantial livestock feed production needed (43, 50, 51). As consumers in the Q5 of the provegetarian score ate less meat, especially ruminant meat, compared to participants in the other quintiles, the difference in GHG emissions is further increased. A previous study showed that a diet in which ruminant meat is replaced by monogastric meat (pork or poultry) reduced GHG emissions from 20 to 35% and land-use impacts from 30 to 50% (50).

In another study, the CED was computed at the farm level using the LCA method, and it was shown that a 60% decrease in daily meat consumption that is replaced by planted-based products led to an up to 38% decrease in CED, according to various scenarios of self-sufficiency in Austria (48). The review by Perignon et al., which covers 10 cohort studies on the environmental impact of observed individual diets, demonstrates that low-meat diets are more environmentally friendly (43).

Livestock farming requires a substantial input of fossil energy due to farm facilities and production of feed (3). Moreover, plant products have higher energy efficiency when considering the ratio of outputs/inputs for each calorie (52). Regarding the CED by food group and by quintile, there is no clear difference in the CED contribution between ruminant meat and monogastric meat. Considering the level of consumption, food group contribution to CED is probably driven more by the difference in intake than energy use for the different types of meat since the differences they are less noticeable than for GHG emissions (53).

Finally, similar results on land use were found when the average Danish diet was replaced by the new Nordic diet containing 35% less meat with a 24% decrease in diet-related land use. In the model performed for the EPIC-Netherlands cohort, the substitution of meat with 35 g/d of plant products led to an up to 12% decrease in land use (49). Moreover, the review of Hallström et al., which included 14 original studies (mainly based on modeling methods), showed that vegan diets reduced land use up to 60 and 50% for men and women, respectively (50). In fact, livestock farming is the largest user of land due to the total area need for grazing and feed crop production (5).

It is worth noting that beyond the benefits to the environment, diets rich in plant products also provide important nutritional and health benefits (54, 55).

We showed that introducing organic food to one’s diet had a significant positive environmental effect on GHG emissions in only diets rich in plant products. However, when considering a diet with a moderate amount of plant products, this effect was not substantial.

The weak moderating effect of the organic consumption in a diet with a moderate amount of plant products can be explained by several hypotheses. First, no difference in GHG emissions was reported for both conventional and organic beef and milk production systems (20). In addition, GHG emissions from chicken and pork organic farming practices are higher because feed production is more substantial due to a longer cycle of production and a lower growth rate (in relation to a lower feed-efficiency conversion) (20). Moreover, GHG emissions from organic pork farming practices can be higher because of the high level of nitrous oxide emissions from straw litter (19). However, the differences between chicken and pork production systems have not yet been consistently measured, and further research is needed to improve the reliability of calculating GHG emissions for different farming practices. Second, organic farming results in lower GHG emissions when emissions are expressed by units of area, and no clear trends emerge when they are expressed by units of product weight (18). Finally, organic practices have obvious beneficial effects on GHG emissions in terms of plant production because of the exclusion of synthetic fertilizers that result in high N_2_O and CO_2_ emissions (19, 56). Finally, the proportion of organic food consumption in the diet may be too low in the first provegetarian score quintiles to detect differences in GHG emissions. Considering the CED indicator, the ratio of organic food in the diet positively affects diet-related environmental impacts with increasing effects across provegetarian score quintiles. Organic practices prohibit the use of synthetic fertilizers which induce high costs in energy for their production and require the use of less mineral fertilizers and feed concentrates (56). However, some studies have determined that CED can be up to 40% higher in organic farming than in conventional systems (19). Another explanation relies on the fact that among the high consumers of organic foods, plant-based food consumption was higher overall. However, the correlation coefficient between the provegetarian score and the level of organic food consumption was estimated to be 0.4.

Regarding land occupation, the level of organic food consumption had a positive impact on diets rich in plant products and had no impact on diets with moderate level of plant product intake. These findings are noteworthy since organic systems require relatively more land (20, 56, 57) than conventional production systems. These lower crop yields are due to lower total nitrogen inputs per hectare (20). Our results may be explained by the fact that in the Q5 of the provegetarian score, consumers that eat a substantial amount of organic food exhibited higher plant-based consumption than their conventional counterparts and thus may have exhibited a lower consumption of meat. Moreover, according to Pimentel and Pimentel, grains and some legumes, which were highly consumed by participants in the Q5 of the provegetarian score, are produced more efficiently than fruits and vegetables (42). This may have led to a reduction in the negative impact of organic production on plant production yields. The absence of a differential effect of organic food consumption on land use for a diet with a moderate amount of plant products may be related to the fact that the ratio of organic foods in the diet is too low to detect any association, which is the same for GHG emissions. These findings regarding land occupation need further investigation since future improvements of management techniques and crop varieties may reduce the difference in crop yields between organic and conventional systems (9). Although this was not evaluated in our study, organic systems generally offer environmental services, do not use pesticides, increase resilience of agriculture and can mitigate the future effects of climate change on yields (58).

The limitations of this study should be noted. An extrapolation of these results to the general population should be done with caution as the participants who completed the BioNutriNet questionnaires were probably more concerned with nutrition and health-related issues. It should be noted that the percentage of participants with a very high consumption of organic foods, as observed in our study, is likely to be minimal in France. The use of a food frequency questionnaire may be prone to incorrectly estimating habitual diets, which is similar to other self-reported food consumption tools (59). Moreover, the effects of the systems of production on the environment should be considered with caution. Indeed, among similar systems of production, effects can be largely different due to climate conditions, soil types and farm management (18, 56). Other indicators such as pesticide use, leaching, and soil quality would have been relevant to addressing the environmental impacts of production systems (60, 61). In addition, our data included neither the origin nor the seasonality of food products, which may impact environmental assessments. Furthermore, environmental impacts were assessed at the farm level and did not consider all of the production, transformation and distribution stages.

However, our study also presents notable strengths. First, to the best of our knowledge, this is the first study to distinguish production modes in the assessment of food consumption and several subsequent environmental impacts. This is also the first study to investigate moderating effects of organic food consumption on the environmental impact of observed diets. Modeling studies do not necessarily consider isocaloric or representative substitutions. For example, replacing meat with fruit and legumes may not appear entirely realistic. Meat would probably be replaced by energy-dense products such as cereals, potatoes, and legumes. Moreover, these modeling studies rely on small cohorts. Therefore, it was crucial to focus on actual diets assessed in a large cohort to confirm or refute the results from modeling studies. Concerning the strengths of this study, our study is based on a large sample, which allows a wide diversity of dietary behaviors to be considered and in particular eco-friendly behaviors, using accurate environmental and consumption data. The provegetarian score also presents several advantages when compared to other dietary indexes commonly used in the literature such as the Mediterranean diet score (62). Indeed, while the Mediterranean diet recommends limiting milk and red meat, it also recommends consuming fish even though a major part of the fishing industry is not sustainable (63) and degrades maritime ecosystem functions by altering the food chain and fish habitats (64). Finally, the provegetarian score reflects different emerging dietary patterns (e.g., flexitarian diets) that tend to reduce consumption of animal products.

In conclusion, diet-related GHG emissions, CED, and land occupation indicators are negatively associated with a plant-based diet, regardless of the level of organic food consumption. Furthermore, the consumption of organic food showed additional beneficial impacts only in diets rich in plant products. This study demonstrates that the environmental impacts of diets should not only be evaluated in terms of dietary patterns but also should integrate production systems.

## Ethics Statement

The design was conducted according to the guidelines laid down in the Declaration of Helsinki and was approved by the Institutional Review Board of the French Institute for Health and Medical Research (IRB INSERM no. 0000388FWA00005831) and the “Commission Nationale de l’Informatique et des Libertés” (CNIL no. 908450 and no. 909216). All participants signed an electronic informed consent.

## Author Contributions

EK-G, SH, PP, and DL designed the research; CL, LS, BA, BL, PP, DL, JB, and EK-G conducted the research; CL, LS, BA, JB, and EK analyzed the data; and CL and EK wrote the paper. CL, LS, BA, BL, PP, DL, JB, and EK were involved in interpreting the results and editing the manuscript. CL, LS, and EK had primary responsibility for the final content. All authors read and approved the final manuscript.

## Conflict of Interest Statement

The authors declare that the research was conducted in the absence of any commercial or financial relationships that could be construed as a potential conflict of interest.

## Abbreviations

GHG, greenhouse gas; CED, cumulative energy demand; LCA, life cycle analysis; Q, quintile; PUFA, polyunsaturated acid; MUFA, monounsaturated acid; SFA, saturated fatty acid.

## References

[1] Fischer CG Garnett T Plates, Pyramids and Planets Developments in National Healthy and Sustainable Dietary Guidelines: A State of Plays Assessment. Oxford, UK: Food and Agriculture Organization of the United Nations and Food Climate Research Network (2016).

[2] Birt C Buzeti T Grosso G Justesen L Lachat C Lafranconi A Healthy and Sustainable Diets for European Countries. Utrecht: European Public Health Association (EUPHA) (2017).

[3] Tackling Climate Change through Livestock: A Global Assessment of Emissions and Mitigation Opportunities – i3437e.pdf. (2017). Available from: http://www.fao.org/docrep/018/i3437e/i3437e.pdf

[4] World Agriculture: Towards 2015/2030 A FAO PERSPECTIVE. (2017). Available from: http://www.fao.org/3/a-y4252e.pdf

[5] Livestock’s Long Shadow Environmental Issues and Options. (2017). Available from: ftp://ftp.fao.org/docrep/fao/010/a0701e/a0701e.pdf

[6] Garnett T Where are the best opportunities for reducing greenhouse gas emissions in the food system (including the food chain)? Food Policy (2011) 36(Suppl 1):S23–32. 10.1016/j.foodpol.2010.10.010

[7] Westhoek H Lesschen JP Rood T Wagner S De Marco A Murphy-Bokern D Food choices, health and environment: effects of cutting Europe’s meat and dairy intake. Glob Environ Change (2014) 26:196–205. 10.1016/j.gloenvcha.2014.02.004

[8] Organic Agriculture: What Is Organic Agriculture? (2017). Available from: http://www.fao.org/organicag/oa-faq/oa-faq1/en/

[9] Reganold JP Wachter JM. Organic agriculture in the twenty-first century. Nat Plants (2016) 2:15221. 10.1038/nplants.2015.221 27249193

[10] Scialabba N Hattam C Organic agriculture, environment and food security. Food Agric Org (2002):264.

[11] Lotter DW Organic agriculture. J Sustain Agric (2003) 21(4):59–128. 10.1300/J064v21n04_06

[12] Tuck SL Winqvist C Mota F Ahnström J Turnbull LA Bengtsson J. Land-use intensity and the effects of organic farming on biodiversity: a hierarchical meta-analysis. J Appl Ecol (2014) 51(3):746–55. 10.1111/1365-2664.12219 25653457 PMC4299503

[13] Connor DJ Mínguez MI Evolution not revolution of farming systems will best feed and green the world. Glob Food Secur (2012) 1:106–13.

[14] Kirchmann H Thorvaldsson G Challenging targets for future agriculture. Eur J Agron (2000) 12(3–4):145–61. 10.1016/S1161-0301(99)00053-2

[15] Dalgaard T Halberg N Porter JR A model for fossil energy use in Danish agriculture used to compare organic and conventional farming. Agric Ecosyst Environ (2001) 87(1):51–65. 10.1016/S0167-8809(00)00297-8

[16] Lynch DH Halberg N Bhatta GD Environmental impact of organic agriculture in temperate regions. CAB Rev. (2012). Available from: http://orgprints.org/20725/

[17] Lee KS Choe YC Park SH. Measuring the environmental effects of organic farming: a meta-analysis of structural variables in empirical research. J Environ Manage (2015) 162:263–74. 10.1016/j.jenvman.2015.07.021 26254994

[18] Koen M Aertsens J Van Huylenbroeck G A meta-analysis of the differences in environmental impacts between organic and conventional farming. Br Food J (2009) 111(10):1098–119. 10.1108/00070700910992925

[19] Tuomisto HL Hodge ID Riordan P Macdonald DW Does organic farming reduce environmental impacts? A meta-analysis of European research. J Environ Manage (2012) 112:309–20. 10.1016/j.jenvman.2012.08.018 22947228

[20] Treu H Nordborg M Cederberg C Heuer T Claupein E Hoffmann H Carbon footprints and land use of conventional and organic diets in Germany. J Clean Prod (2017) 161:127–42. 10.1016/j.jclepro.2017.05.041

[21] Baudry J Méjean C Allès B Péneau S Touvier M Hercberg S Contribution of organic food to the diet in a large sample of French adults (the NutriNet-Santé Cohort Study). Nutrients (2015) 7(10):8615–32. 10.3390/nu7105417 26506372 PMC4632437

[22] Aleksandrowicz L Green R Joy EJM Smith P Haines A. The impacts of dietary change on greenhouse gas emissions, land use, water use, and health: a systematic review. Wiley AS, editor. PLoS One (2016) 11(11):e0165797. 10.1371/journal.pone.0165797 27812156 PMC5094759

[23] Rosi A Mena P Pellegrini N Turroni S Neviani E Ferrocino I Environmental impact of omnivorous, ovo-lacto-vegetarian, and vegan diet. Sci Rep (2017) 7(1):6105. 10.1038/s41598-017-06466-8 28733610 PMC5522483

[24] Fiddes N Meat: A Natural Symbol. London: Routledge (1991). 260 p.

[25] Martínez-González MA Sánchez-Tainta A Corella D Salas-Salvadó J Ros E Arós F A provegetarian food pattern and reduction in total mortality in the Prevención con Dieta Mediterránea (PREDIMED) study. Am J Clin Nutr (2014) 100(Suppl 1):320S–8S. 10.3945/ajcn.113.071431 24871477

[26] Hercberg S Castetbon K Czernichow S Malon A Mejean C Kesse E The Nutrinet-Santé Study: a web-based prospective study on the relationship between nutrition and health and determinants of dietary patterns and nutritional status. BMC Public Health (2010) 10:242 10.1186/1471-2458-10-242 20459807 PMC2881098

[27] INSEE. Définition – Unité de consummation. Insee (2017). Available from: https://www.insee.fr/fr/metadonnees/definition/c1802

[28] Craig CL Marshall AL Sjöström M Bauman AE Booth ML Ainsworth BE International physical activity questionnaire: 12-country reliability and validity. Med Sci Sports Exerc (2003) 35(8):1381–95. 10.1249/01.MSS.0000078924.61453.FB 12900694

[29] Hallal PC Victora CG Reliability and validity of the International Physical Activity Questionnaire (IPAQ). Med Sci Sports Exerc (2004) 36(3):556 10.1249/01.MSS.0000117161.66394.07 15076800

[30] Hagströmer M Oja P Sjöström M. The International Physical Activity Questionnaire (IPAQ): a study of concurrent and construct validity. Public Health Nutr (2006) 9(6):755–62. 10.1079/PHN2005898 16925881

[31] Alcohol Guidelines Review – Report from the Guidelines Development Group to the UK Chief Medical Officers. (2017). Available from: https://www.gov.uk/government/uploads/system/uploads/attachment_data/file/545739/GDG_report-Jan2016.pdf

[32] Kesse-Guyot E Castetbon K Touvier M Hercberg S Galan P. Relative validity and reproducibility of a food frequency questionnaire designed for French adults. Ann Nutr Metab (2010) 57(3–4):153–62. 10.1159/000321680 21079389

[33] Table de composition des aliments – NutriNet-Santé, Serge Hercberg. (2017). Available from: https://www.decitre.fr/livres/table-de-composition-des-aliments-9782717865370.html

[34] Verger EO Mariotti F Holmes BA Paineau D Huneau J-F. Evaluation of a diet quality index based on the probability of adequate nutrient intake (PANDiet) using National French and US Dietary Surveys. PLoS One (2012) 7(8):e42155. 10.1371/journal.pone.0042155 22870293 PMC3411671

[35] Chauliac M Razanamahefa L Choma C Boudot J Houssin D [National health and nutrition program: challenges of a global action plan]. Rev Prat (2009) 59(1):10–2. 19253871

[36] Solagro. Dialecte. Solagro (2017). Available from: https://solagro.org/nos-travaux-et-productions

[37] Basset-Mens C Small B Paragahawewa UH Langevin B Paula B Life cycle thinking and sustainable food production. Int J Prod Lifecycle Manage (2009) 4(1–3):252–69. 10.1504/IJPLM.2009.031675

[38] Owens JW Life-cycle assessment in relation to risk assessment: an evolving perspective. Risk Anal (1997) 17(3):359–65. 10.1111/j.1539-6924.1997.tb00874.x

[39] Implementation of Life Cycle Impact Assessment Methods. (2017). Available from: http://www.proyectaryproducir.com.ar/public_html/Seminarios_Posgrado/Material_de_referencia/EcoInvent%2003_LCIA-Implementation-v2.2.pdf

[40] Willett WC Howe GR Kushi LH. Adjustment for total energy intake in epidemiologic studies. Am J Clin Nutr (1997) 65(4):1220S–8S. 10.1093/ajcn/65.4.1220S 9094926

[41] Schofield WN. Predicting basal metabolic rate, new standards and review of previous work. Hum Nutr Clin Nutr (1985) 39(Suppl 1):5–41. 4044297

[42] Pimentel D Pimentel M. Sustainability of meat-based and plant-based diets and the environment. Am J Clin Nutr (2003) 78(3):660S–3S. 12936963 10.1093/ajcn/78.3.660S

[43] Perignon M Vieux F Soler L-G Masset G Darmon N. Improving diet sustainability through evolution of food choices: review of epidemiological studies on the environmental impact of diets. Nutr Rev (2017) 75(1):2–17. 10.1093/nutrit/nuw043 27974596 PMC5155614

[44] Auestad N Fulgoni VL. What current literature tells us about sustainable diets: emerging research linking dietary patterns, environmental sustainability, and economics. Adv Nutr (2015) 6(1):19–36. 10.3945/an.114.005694 25593141 PMC4288277

[45] Scarborough P Appleby PN Mizdrak A Briggs ADM Travis RC Bradbury KE Dietary greenhouse gas emissions of meat-eaters, fish-eaters, vegetarians and vegans in the UK. Clim Change (2014) 125(2):179–92. 10.1007/s10584-014-1169-1 25834298 PMC4372775

[46] Tilman D Clark M. Global diets link environmental sustainability and human health. Nature (2014) 515(7528):518–22. 10.1038/nature13959 25383533

[47] Macdiarmid JI Kyle J Horgan GW Loe J Fyfe C Johnstone A Sustainable diets for the future: can we contribute to reducing greenhouse gas emissions by eating a healthy diet? Am J Clin Nutr (2012) 96(3):632–9. 10.3945/ajcn.112.038729 22854399

[48] Fazeni K Steinmüller H Impact of changes in diet on the availability of land, energy demand, and greenhouse gas emissions of agriculture. Energy Sustain Soc (2011) 1(1):6 10.1186/2192-0567-1-6

[49] Biesbroek S Bueno-de-Mesquita HB Peeters PHM Verschuren WM van der Schouw YT Kramer GFH Reducing our environmental footprint and improving our health: greenhouse gas emission and land use of usual diet and mortality in EPIC-NL: a prospective cohort study. Environ Health (2014) 13(1):27. 10.1186/1476-069X-13-27 24708803 PMC4013533

[50] Hallström E Carlsson-Kanyama A Börjesson P Environmental impact of dietary change: a systematic review. J Clean Prod (2015) 91:1–11. 10.1016/j.jclepro.2014.12.008

[51] Bryngelsson D Hedenus F Johansson DJA Azar C Wirsenius S How do dietary choices influence the energy-system cost of stabilizing the climate? Energies (2017) 10(2):182 10.3390/en10020182

[52] Eshel G Martin PA Diet, energy, and global warming. Earth Interact (2006) 10(9):1–17. 10.1175/EI167.1

[53] de Vries M de Boer IJM Comparing environmental impacts for livestock products: a review of life cycle assessments. Livest Sci (2010) 128(1–3):1–11. 10.1016/j.livsci.2009.11.007

[54] Schwingshackl L Schwedhelm C Hoffmann G Lampousi A-M Knüppel S Iqbal K Food groups and risk of all-cause mortality: a systematic review and meta-analysis of prospective studies. Am J Clin Nutr (2017) 105(6):1462–73. 10.3945/ajcn.117.153148 28446499

[55] Springmann M Godfray HCJ Rayner M Scarborough P. Analysis and valuation of the health and climate change cobenefits of dietary change. Proc Natl Acad Sci U S A (2016) 113(15):4146–51. 10.1073/pnas.1523119113 27001851 PMC4839446

[56] Gomiero T Pimentel D Paoletti MG Environmental impact of different agricultural management practices: conventional vs. organic agriculture. Crit Rev Plant Sci (2011) 30(1–2):95–124. 10.1080/07352689.2011.554355

[57] Baroni L Cenci L Tettamanti M Berati M Evaluating the environmental impact of various dietary patterns combined with different food production systems. Eur J Clin Nutr (2006) 61(2):279–86. 10.1038/sj.ejcn.1602522 17035955

[58] Building Resilience for an Unpredictable Future: How Organic Agriculture Can Help Farmers Adapt to Climate Change. (2017). Available from: http://www.fao.org/3/a-ah617e.pdf

[59] Cade J Thompson R Burley V Warm D. Development, validation and utilisation of food-frequency questionnaires – a review. Public Health Nutr (2002) 5(4):567–87. 10.1079/PHN2001318 12186666

[60] Gattinger A Muller A Haeni M Skinner C Fliessbach A Buchmann N Enhanced top soil carbon stocks under organic farming. Proc Natl Acad Sci U S A (2012) 109(44):18226–31. 10.1073/pnas.1209429109 23071312 PMC3497757

[61] Skinner C Gattinger A Muller A Mäder P Flieβbach A Stolze M Greenhouse gas fluxes from agricultural soils under organic and non-organic management – a global meta-analysis. Sci Total Environ (2014) 468–469:553–63. 10.1016/j.scitotenv.2013.08.098 24061052

[62] Trichopoulou A Costacou T Bamia C Trichopoulos D Adherence to a Mediterranean diet and survival in a Greek population. N Engl J Med (2003) 348(26):2599–608. 10.1056/NEJMoa025039 12826634

[63] Pauly D Christensen V Guénette S Pitcher TJ Sumaila UR Walters CJ Towards sustainability in world fisheries. Nature (2002) 418(6898):689–95. 10.1038/nature01017 12167876

[64] Dayton PK Thrush SF Agardy MT Hofman RJ Environmental effects of marine fishing. Aquat Conserv (1995) 5(3):205–32. 10.1002/aqc.3270050305

