# Corrigendum: Environmental Impacts of Plant-Based Diets: How Does Organic Food Consumption Contribute to Environmental Sustainability?

**DOI:** 10.3389/fnut.2018.00026

**Published:** 2018-04-18

**Authors:** Camille Lacour, Louise Seconda, Benjamin Allès, Serge Hercberg, Brigitte Langevin, Philippe Pointereau, Denis Lairon, Julia Baudry, Emmanuelle Kesse-Guyot

**Affiliations:** ^1^Equipe de Recherche en Epidémiologie Nutritionnelle (EREN), Centre d'Epidémiologie et Statistiques Sorbonne Paris Cité, INSERM (U1153), INRA (U1125), CNAM, Université Paris 13, COMUE Sorbonne Paris Cité, obigny, France; ^2^Agence de l'Environnement et de la Maîtrise de l'Energie, Angers, France; ^3^Département de Santé Publique, Hôpital Avicenne, Bobigny, France; ^4^Solagro, Toulouse, France; ^5^Nutrition Obésité et Risque Thrombotique (NORT), Aix Marseille Université, INRA 1260, INSERM UMR S 1062, Marseille, France

**Keywords:** provegetarian dietary pattern, organic food consumption, eco-friendly farming, diet-related environmental impact, sustainability

There was a mistake in the values of the first column of Table 4 as published. The correct version of Table 4 appears below. The authors apologize for this mistake. This error does not change the scientific conclusions of the article in any way.

**Table 4:** Association between provegetarian score tertile and environmental impacts according to the level of organic food consumption, BioNutriNet study, 2014.

	**Overall**		**Level of contribution of organic food to the diet**						
			**Low (0.03)**		**Medium (0.23)**		**High (0.63)**		
**GHG emissions (CO2eq/d)**	**Mean^a^**	**95%CL**	**Mean^a^**	**95%CL**	**Mean^a^**	**95%CL**	**Mean^a^**	**95%CL**	
Q1 provegetarian score	4.56	(4.51–4.60)	4.59	(4.53–4.65)	4.56	(4.48–4.63)	4.10	(3.99–4.22)	
Q2 provegetarian score	4.05	(4.01–4.08)	4.13	(4.08–4.18)	4.05	(4.00–4.10)	3.74	(3.66–3.81)	
Q3 provegetarian score	3.62	(3.62–3.66)	3.73	(3.68–3.78)	3.68	(3.63–3.74)	3.34	(3.28–3.41)	
Q4 provegetarian score	3.23	(3.20–3.27)	3.45	(3.39–3.51)	3.38	(3.33–3.43)	2.94	(2.89–2.99)	
Q5 provegetarian score	2.27	(1.33–2.29)	2.93	(2.87–2.99)	2.72	(2.67–2.76)	2.12	(2.09–2.14)	
*P*^b^ interaction									<0.0001
*P*^c^ Q1 vs. Q2									0.9711
*P*^c^ Q1 vs. Q3									0.2764
*P*^c^ Q1 vs. Q4									<0.0001
*P*^c^ Q1 vs. Q5									<0.0001
**Cumulative energy demand (MJ/d)**	**Mean**^a^	**95%CL**	**Mean**^a^	**95%CL**	**Mean**^a^	**95%CL**	**Mean**^a^	**95%CL**	
Q1 provegetarian score	18.55	(18.43–18.67)	18.58	(18.40–18.75)	18.58	(18.39–18.78)	17.33	(17.05–17.63)	
Q2 provegetarian score	17.43	(17.33–17.53)	17.62	(17.47–17.77)	17.47	(17.32–17.63)	16.53	(16.32–16.73)	
Q3 provegetarian score	16.48	(15.52–16.58)	16.87	(16.70–17.04)	16.62	(16.47–16.78)	15.59	(15.41–15.77)	
Q4 provegetarian score	15.62	(15.52–15.73)	16.42	(16.21–16.63)	16.10	(15.93–16.27)	14.62	(14.45–14.78)	
Q5 provegetarian score	13.29	(13.21–13.37)	15.56	(15.33–15.79)	14.72	(14.56–14.89)	12.66	(12.56–12.76)	
*P*^b^ interaction									<0.0001
*P*^c^ Q1 vs. Q2									0.9417
*P*^c^ Q1 vs. Q3									0.1044
*P*^c^ Q1 vs. Q4									<0.0001
*P*^c^ Q1 vs. Q5									<0.0001
**Land occupational (m2/d)**	**Mean**^a^	**95%CL**	**Mean**^a^	**95%CL**	**Mean**^a^	**95%CL**	**Mean**^a^	**95%CL**	
Q1 provegetarian score	11.33	(11.14–11.41)	10.94	(10.78–11.10)	11.58	(11.39–11.78)	11.66	(11.36–11.96)	
Q2 provegetarian score	10.26	(10.17–10.35)	9.89	(9.76–10.03)	10.31	(10.17–10.45)	10.64	(10.45–10.85)	
Q3 provegetarian score	9.34	(9.26–9.43)	8.95	(8.81–9.09)	9.43	(9.29–9.57)	9.61	(9.44–9.79)	
Q4 provegetarian score	8.51	(8.42–8.60)	8.26	(8.10–8.43)	8.68	(8.54–8.83)	8.50	(8.35–8.65)	
Q5 provegetarian score	6.63	(6.57–6.69)	7.03	(6.87–7.19)	7.09	(6.97–7.21)	6.49	(6.41–6.57)	
*P*^b^ interaction									<0.0001
*P*^c^ Q1 vs. Q2									0.7782
*P*^c^ Q1 vs. Q3									0.9696
*P*^c^ Q1 vs. Q4									0.0111
*P*^c^ Q1 vs. Q5									<0.0001

*GHG, Greenhouse gas. Models are adjusted on sex, age, and energy intake*.

a *Adjusted mean were obtained with ANOVA models by level of organic food contribution in the diet. P-trend across the provegetarian score quintile are all < 0.0001 and were obtained with linear contrast test by level of organic food contribution in the diet*.

b *P for interaction between provegetarian score quintiles and the level contribution of organic food to the diet*.

c P-linear trend of Q^*^v.Q1 of provegetarian score. It reflects the linearity of the difference between the 1st and the others quintiles of provegetarian score across the level of organic consumption.

The original article has been updated.

## Conflict of interest statement

The authors declare that the research was conducted in the absence of any commercial or financial relationships that could be construed as a potential conflict of interest.

