# Supplementary material for: Environmental Impacts of Plant-Based Diets: How Does Organic Food Consumption Contribute to Environmental Sustainability?
Source: Front Nutr. 2018 Feb 9;5:8. doi: 10.3389/fnut.2018.00008 (PMC5811770; doi:10.3389/fnut.2018.00008)
Supplement: Supplementary file 1 [file Table_1.PDF]

Supplemental table 1: Food groups of the provegetarian score.

Provegetarian score components	Included foods (FFQ items)
**Plant food groups by quintile**	
**Vegetables**	Avocado, artichoke, onion, garlic, mushroom, salad, carrot, celery, tomato, beetroot, red cabbage, white cabbage, green cabbage, Brussels sprout, cauliflower, broccoli, green bean, chicory, spinach, cucumber, sweet pepper, leek, fennel, pumpkin, turnip, peas, corn, seaweed, soup
**Fruits**	Apple, pear, banana, peach, citrus fruit, strawberry, apricot, melon, raspberry, plum, kiwi, grape, pineapple, mango, lychee, exotic fruit, compote, fruit jus, pure fruit jus, stewed fruit, dried fruit
**Legumes**	Dried vegetables
**Cereals**	White bread, crispbread, cereal, muesli, semolina, quinoa, white rice, wild rice, pasta, wholewheat bread, wholewheat pasta, whole grain rice, sprouted seed, brewer’s yeast
**Potatoes**	Potatoes, jerusalem artichoke
**Nuts**	Oleaginous, salted oleaginous
**Oil**	Sunflower oil, olive oil, groundnut oil, rapeseed oil, corn oil, soybean oil, walnut oil, hazelnut oil, seed oil, sesame oil, coconut oil, linseed oil, safflower oil
**Animal food groups by reverse quintile**	
**Meats and meat products**	Beef, calf, lamb, rabbit, pork, turkey, liver, tripe, breaded meat, sausage, cured ham, cooked ham, saveloy, mortadella, pâté, rillettes, bacon
**Animal fats for cooking or as spread**	Butter
**Eggs**	Eggs
**Fish and other seafood**	Oily fish, white fish, breaded fish, shellfish, crustacean
**Dairy product**	Fresh cream, low-fat fresh cream, , whole milk, semi-skimmed milk, skimmed-milk, fermented milk, plain yogurt, semi-skimmed yogurt, skimmed-yogurt, plain fruit yogurt, semi-skimmed fruit yogurt, bifidus yogurt, cream cheese, cheese, Gouda, goat’s cheese, sheep’s cheese, edam, mozzarella, brie cheese, blue cheese, ice cream
