# Supplementary material for: Environmental Impacts of Plant-Based Diets: How Does Organic Food Consumption Contribute to Environmental Sustainability?
Source: Front Nutr. 2018 Feb 9;5:8. doi: 10.3389/fnut.2018.00008 (PMC5811770; doi:10.3389/fnut.2018.00008)
Supplement: Supplementary file 2 [file Table_2.PDF]

Supplemental table 2: Association between provegetarian score quintile and environmental impacts, by the level of organic food consumption, BioNutriNet Study, 2014

	Level of contribution of organic food to the diet					
	Low (0,03)		Medium (0,23)		High (0,63)	
**GHG emissions (CO_2eq/d)**	Mean[1]	95%CL	Mean[1]	95%CL	Mean[1]	95%CL
Q1 provegetarian score	5,09	[5,01-5,18]	5,14	[5,03-5,24]	4,66	[4,51-4,82]
Q2 provegetarian score	4,04	[3,97-4,1]	4,05	[3,98-4,12]	3,80	[3,71-3,9]
Q3 provegetarian score	3,54	[3,47-3,61]	3,48	[3,41-3,55]	3,28	[3,2-3,36]
Q4 provegetarian score	3,22	[3,14-3,29]	3,21	[3,14-3,27]	2,83	[2,83-2,9]
Q5 provegetarian score	2,94	[2,86-3,02]	2,71	[2,65-2,77]	2,10	[2,1-2,13]
P interaction[2]						<0,0001
P[3] Q1 vs Q2						0,48
P[3] Q1 vs Q3						0,64
P[3] Q1 vs Q4						0,00
P[3] Q1 vs Q5						<0,0001
**Cumulative energy demand (MJ/d)**	Mean[1]	95%CL	Mean[1]	95%CL	Mean[1]	95%CL
Q1 provegetarian score	20,55	[20,26-20,84]	20,82	[20,49-21,16]	19,53	[19,06-20,02]
Q2 provegetarian score	17,26	[17,03-17,49]	17,50	[17,26-17,74]	16,78	[16,48-17,09]
Q3 provegetarian score	16,03	[15,78-16,27]	15,77	[15,53-16]	15,29	[15,03-15,56]
Q4 provegetarian score	15,35	[15,06-15,65]	15,32	[15,07-15,57]	14,11	[13,88-14,35]
Q5 provegetarian score	15,57	[15,21-15,92]	14,63	[14,38-14,89]	12,56	[12,41-12,71]
P interaction[2]						<0,0001
P[3] Q1 vs Q2						0,38
P[3] Q1 vs Q3						0,58
P[3] Q1 vs Q4						0,00
P[3] Q1 vs Q5						<0,0001
**Land use (m^2/d)**	Mean	95%CL	Mean	95%CL	Mean	95%CL
Q1 provegetarian score	12,15	[11,92-12,37]	13,05	[12,77-13,33]	13,25	[12,83-13,68]
Q2 provegetarian score	9,67	[9,5-9,84]	10,31	[10,13-10,5]	10,82	[10,57-11,08]
Q3 provegetarian score	8,48	[8,31-8,65]	8,91	[8,73-9,09]	9,41	[9,2-9,63]
Q4 provegetarian score	7,71	[7,52-7,91]	8,25	[8,07-8,43]	8,18	[8-8,36]
Q5 provegetarian score	7,06	[6,85-7,27]	7,06	[6,9-7,23]	6,44	[6,34-6,54]
P interaction[2]						<0,0001
P[3] Q1 vs Q2						0,38
P[3] Q1 vs Q3						0,74
P[3] Q1 vs Q4						0,08
P[3] Q1 vs Q5						<0,0001

Models are not adjusted. 1 Means were obtained with ANOVA models by level of organic food contribution in the diet. P-trend across the quintile of provegetarian score are all <0.0001 and were obtained with linear contrast test by level of organic food contribution in the diet.2 P for interaction between quintiles of provegetarian score and the level contribution of organic food to the diet. 3P-linear trend of Q versus.Q1 of provegetarian score reflecting the linearity of the difference between the 1st and the others quintiles of provegetarian score across the level of organic consumption.*
