# Supplementary material for: Environmental Impacts of Plant-Based Diets: How Does Organic Food Consumption Contribute to Environmental Sustainability?
Source: Front Nutr. 2018 Feb 9;5:8. doi: 10.3389/fnut.2018.00008 (PMC5811770; doi:10.3389/fnut.2018.00008)
Supplement: Supplementary file 3 [file Presentation_1.PDF]

Supplemental material 1

Supplementary Methods: Description of the methodological approach for the evaluation of environmental impact indicators

Evaluation of the agricultural production stage

1. Data sources and perimeter of analysis

Environmental data were derived from the French diagnostic tool DIALECTE (1). DIALECTE is a comprehensive tool developed by Solagro which aims to describe farming systems and to assess the environmental performance of farms using a global approach at farm scale. To date, DIALECTE database includes information from 2086 farms with various agricultural production systems, in particular organic farming, throughout France. A total of 46% of farms included in the DIALECTE database followed certified organic practices.

Information collected by DIALECTE allowed estimating the environmental impacts at the farm level of 60 raw agricultural products using the Life Cycle Assessment method (LCA). Here, the perimeter of LCA was limited to the agricultural production step. Of note, the upstream processes were included in the assessment such as the production of inputs or energy provision. Conditioning, transport, processing, storage and recycling were excluded from the analysis. This limitation should be considered as relative since most environmental impacts generally occur from “the agricultural phase” (2,3), except alcoholic beverages which require a high level of processing and/or bottling or fruit and vegetables when transported by plane.

The choice of DIALECTE pertained to its great diversity of data based-on real farms from various types of farming systems. Apart from the Agribalyse project (4) in which organic data are scarce, to our knowledge, DIALECTE is to date the only French database that covers such a large panel of data for both organic and conventional agricultural products, making it possible the calculation of LCA-based indicators and for a whole diet.

2. Assessment of environmental indicators at farm scale

Cumulative energy demand (CED)

Cumulative energy demand (in MJ) included consumption of renewable and unrenewable energy as defined by the CED method (5), commonly used in LCA. The calculation system used was based on the Dia’terre® instrument (6). The use of renewable energies could be limited at farm level, as a very low percentage of farms in Dialecte have solar dries or water panels which reduce the consumption of unrenewable energies.

Table 1 shows the different cumulative energy demand items and the nature of the data used. Most of the energy demands positions were evaluated at the farm level; an attribution

(with generic energy demand coefficient) was used to share the energetic consumption between vegetable production and livestock building.

Table 1 : Cumulative energy demand posts and calcultation method by production

Cumulative energy demand posts	Survey data	Attribution
Directs energy (without irrigation)		
Fuel*<br>(Fuel, lubricants, diesel and petrol)<br>Gas<br>(propane, butane, natural gas)<br>Electricity	Data at farm level	Allocation of energy consumption in proportion to the theoretical consumption of crops and livestock.
Direct energy for irrigation		
Fuel*<br>Electricity *	Data at farm level	Prorated calculation of attribution in function of theoretic consumption between vegetable production and livestock building.
Indirect energy		
Livestock and veterinary products<br>Mechanization<br>Building (<30 years)<br>Plastic sheeting<br>Preservatives silage	Data at farm level	Non-inclusion consumption<br>Prorated calculation of attribution for vegetable production, in function of land occupation.
Fertilizer<br>Phytosanitary product	Data at crop level	No necessary
Feed	Data at farm level	Prorated calculation of attribution for animals in function of the consumption of forage and concentrates.

* *including consumption for third parties*

Greenhouse gas emissions (GHGEs)

The GHG referred to the well-known definition established by the IPCC (The Intergovernmental Panel on Climate Change), i.e. the quantity of three GHGs (carbon dioxide CO_2, methane CH_4 and nitrous oxide N_2O), weighted by their 100-year global warming potential, in kg $C0_{2eq}$. Their calculation was based on the methodology developed in the tool Dia'terre®(6).

Table 2 shows the different posts of GHG emissions accounting in the LCA. Attribution (with generic energy demand coefficient) was also used to share the emissions between vegetable production and livestock building.

Table 2: Point of GHG emissions and calculation method by production

Point of GHG emissions	Survey data	Attribution
Direct energy		
Direct energy without irrigation (fuel, Gas, electricity)	Data at farm level	Prorated calculation of attribution in function of theoretic consumption between vegetable production and livestock building
Direct energy for irrigation (fuel, electricity)	Data at farm level	Prorated calculation of attribution in function of water consumption by crop
Indirect energy		
Availability of input, building < 30 years, mechanization, plastic, preservatives silage, and veterinary products	Data at farm level	Prorated calculation of attribution for vegetable production, in function of land occupation.
Availability of fertilizer And phytosanitary product	Data at crop level	N\A
Feed	Data at farm level	Prorated calculation of attribution for animals in function of the consumption of forage and concentrates.
**Fugitive emissions**		
Enteric fermentation	Data by domestic animal category	N\A
Direct emission of N_2O (Spreading nitrogen fertilizers and livestock effluents (via pasture including), mineralization of crop residues.	Data at crop level	
Indirect emission of N_2O (nitrogen atmospheric deposition, runoff and nitrogen leaching)		
**Carbone storage in soil and in trees**		Non-inclusion consumption

Land occupation

Land occupation corresponded to the area that is required to produce raw agricultural products, without taking into account the duration of the land use. It is expressed as the inverse of yield, in $m^2. kg^{-1}$. This indicator differs from the LCA indicator *land use,* expressed in $m^2.kg^{-1}$*year, which reflects the surface of land occupied over a fixed period of time. Both indicators are similar when the production period is 1 year, which corresponds to most agricultural production cycles.

For 29 types of vegetables, the impacts were calculated with the mean yield between 2010 and 2015 of the agricultural annual French statistics, for conventional agriculture. Indeed, most vegetable is produced on small areas and yield calculation over one hectare may lead to over or underestimation. We used a rebate coefficient for organic of 23% for all vegetable crops, excepted for strawberries, 41% and salads 14%(7). For vegetable crops, the impacts per hectare were computed depending on the place of production: heated glasshouses, cold frames and open fields (7).

For animal production, the indicator was calculated as the sum of the areas needed for pasture and feed production including purchased feed (while excluding surfaces related to livestock farm facilities).

3. Aggregation and affectation of some specific products

Some agricultural products available in Dialecte but not in BioNutriNet database have been aggregated into one single product reference (see table 3), while affecting a weighting based on the Annual Agricultural Survey from the French Ministry of Agriculture (8).

Table 3 : Methodoly for aggregation of some specific agricultural prodducts

Aggregated agricultural product	Dialecte agricultural product	Proportion
Olive	Black olive	0.5
	Green olive	0.5
Chicken	Standard chicken	0.88
	Labelled chicken	0.12
Bovine meat	From cattle rearing and fattening farms	0.66
	From dairy cattle	0.34
Sheep meat	Sheep fattening	0.9
	Dairy Sheep	0.1

Tomatoes produced under heated greenhouses were considered as « cherry tomatoes » or « fresh tomatoes » and tomatoes produced in open fields as « cooked tomatoes » and « tomato pulp ».

4. Determination of the reference value

Using the methodology described above, we obtained environmental impacts for 62 raw agricultural products from 2086 agricultural holdings available in DIALECTE, of which 46% were conducted according to organic principles. Regarding organic farming, the number of cases available ranged from 2 (fattened duck) to 355 (beef meat) (IQR: 34; 348). Concerning conventional farming, number of available data ranged from 1 (nuts) to 408 (soft wheat) (IQR: 40; 348). We chose to use the median as the central value as the presence of outliers is relatively common in this type of survey.

For products with a high contribution to the diet and for which data were not available in Dialecte, such as seafood (N=6), honey (N=1), tropical products (N=12) and for some products with few references in Dialecte (N=13), impacts were assessed through literature searches (e.g. using data from Agribalyse database, or using data published in scientific literature).

Finally, we obtained a database with environmental indicators of reference values for 92 raw agricultural products (60 from DIALECTE and 32 from literature searches).

Conversion from raw agricultural products to food items

As our goal was to assess the environmental impacts of diets, we conducted a set of conversions to obtain data at the consumer level.

1. Conversion from raw agricultural products to ingredients

As a first step, to obtain values for each indicators and ingredients from agricultural raw products, economic attributions by co-products were applied followed by the allocations of cooking, and edibility coefficients in order to obtain indicator values for the ingredient as consumed, using data from literature and NutriNet-santé composition database (for allocations of cooking and edibility coefficients).

Environmental impact (ingredient) = Environmental impact (raw agriculture product)*economic attributions factor (in kg)*cooking coefficient*edibility coefficient

59 minor ingredients accounting for more than 5% in at least one recipe were without data. It was true in particular for some food from tropical areas, some alcoholic beverages, grains or yeast. Their environmental impacts were considered as null.

2. Conversion from ingredients to food items

Each of the 264 items of the Org-FFQ (Organic Food Frequency Questionnaire to assess the food consumption in the study) was composed of sub-items (or foods). For example the item pasta was composed of many sub-items (vermicelli, pasta, Chinese noodles, egg pasta, fresh pasta, and egg and fresh pasta spinach flavor). The composition of each item was the result of the multiplication of sex-specific consumption frequency of each sub-item (determined using the R24 tool of NutriNet-Santé) by the ingredients constituting the sub-item. A food item could have several ingredients, so a sum of these ingredients was done.

Overall, a total of 766 ingredients are components of the 264 food items of the Org-FFQ. For feasibility reasons, the environmental impacts of the ingredients which accounted for at least 5% of the recipe were computed, corresponding to 442 ingredients.

About 75% of food items had more than 98.8% of their composition covered by the 442 ingredients. When an ingredient was missing for a given item, environmental impacts were “standardized” for 100g. The ingredients especially concerned by the standardization were fish products, jam, cheeses and dressings.

Finally, we obtained environmental impacts for most of the 264 food items in organic and conventional forms. No data were available for 22 items for cumulative energy demand, 21 items for GHGE and 25 items for land occupation. Missing data included the following food items: water, certain alcoholic beverages, tropical fruit or vegetables, grains and certain oils.

Figure 1 summarizes the different steps carried out for the assessment of environmental impact indicators.

Figure 1: Steps of environmental assessment from the raw agricultural products to items consumed

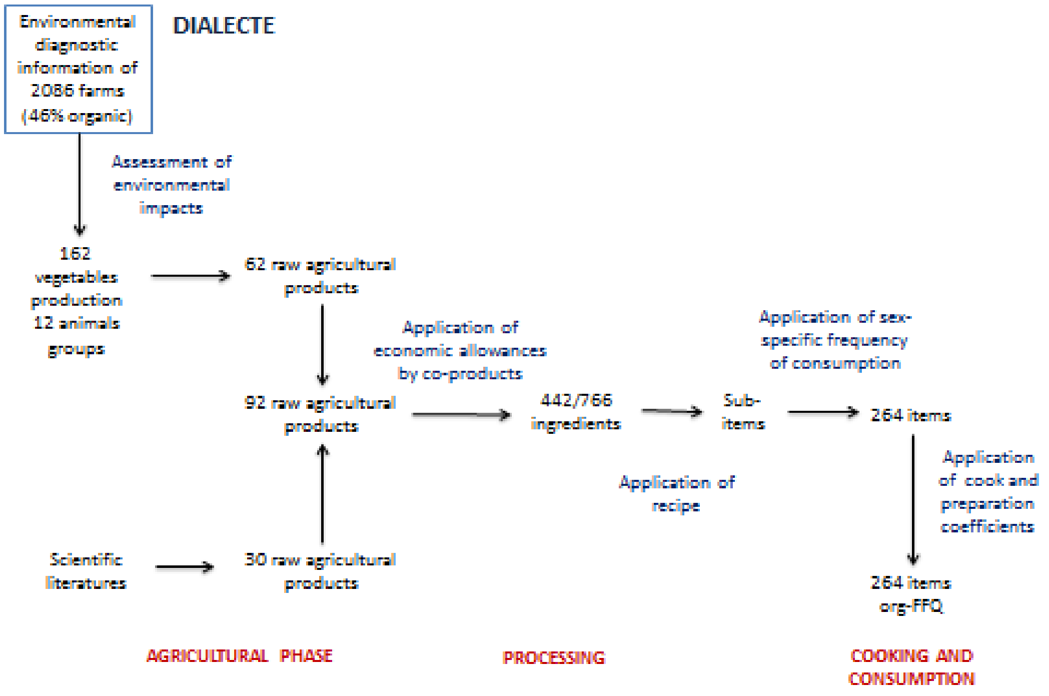

Bibliography

1. Pointereau P, Langevin B, Gimaret M. DIALECTE, a comprehensive and quick tool to assess the agro-environmental performance of farms (PDF Download Available) [Internet]. ResearchGate. 2012 [cited 2017 Jun 16]. Available from: https://www.researchgate.net/publication/265288497_DIALECTE_a_comprehensive_and_quick_tool_to_assess_the_agro-environmental_performance_of_farms

2. Weidema B., Meeusen MJG. Agricultural Data for Life Cycle Assessments. La Hague, Pays-Bas; 2000. 189 p.

3. Clune S, Crossin E, Verghese K. Systematic review of greenhouse gas emissions for different fresh food categories. J Clean Prod. 2017 Jan 1;140, Part 2:766–83.

4. L’évaluation environnementale en agriculture - L’outil AGRIBALYSE® [Internet]. ADEME. [cited 2017 Jun 9]. Available from: http://www.ademe.fr/expertises/produire-autrement/production-agricole/passer-a-laction/dossier/levaluation-environnementale-agriculture/loutil-agribalyser

5. Frischknecht R, Jungbluth N, Althaus H., Bauer C, Doka G, Dones R, et al. Implementation of Life Cycle Impact Assessment Methods Report 3.

6. ADEME. Dia’terre®. Synthèse du guide de la méthode et guide des valeurs. 2015.

7. de Ponti T, Rijk B, van Ittersum MK. The crop yield gap between organic and conventional agriculture. Agric Syst. 2012 Apr;108:1–9.

8. Ministère de l’agriculture et de l’alimentation - agreste - La statistique, l’évaluation et la prospective agricole [Internet]. [cited 2017 Jun 9]. Available from: http://www.agreste.agriculture.gouv.fr/
