# Supplementary material for: Environmental Impacts of Plant-Based Diets: How Does Organic Food Consumption Contribute to Environmental Sustainability?
Source: Front Nutr. 2018 Feb 9;5:8. doi: 10.3389/fnut.2018.00008 (PMC5811770; doi:10.3389/fnut.2018.00008)
Supplement: Supplementary file 4 [file Image_1.PDF]

Supplemental figure 1: Environmental impacts by quintile of provegetarian score.

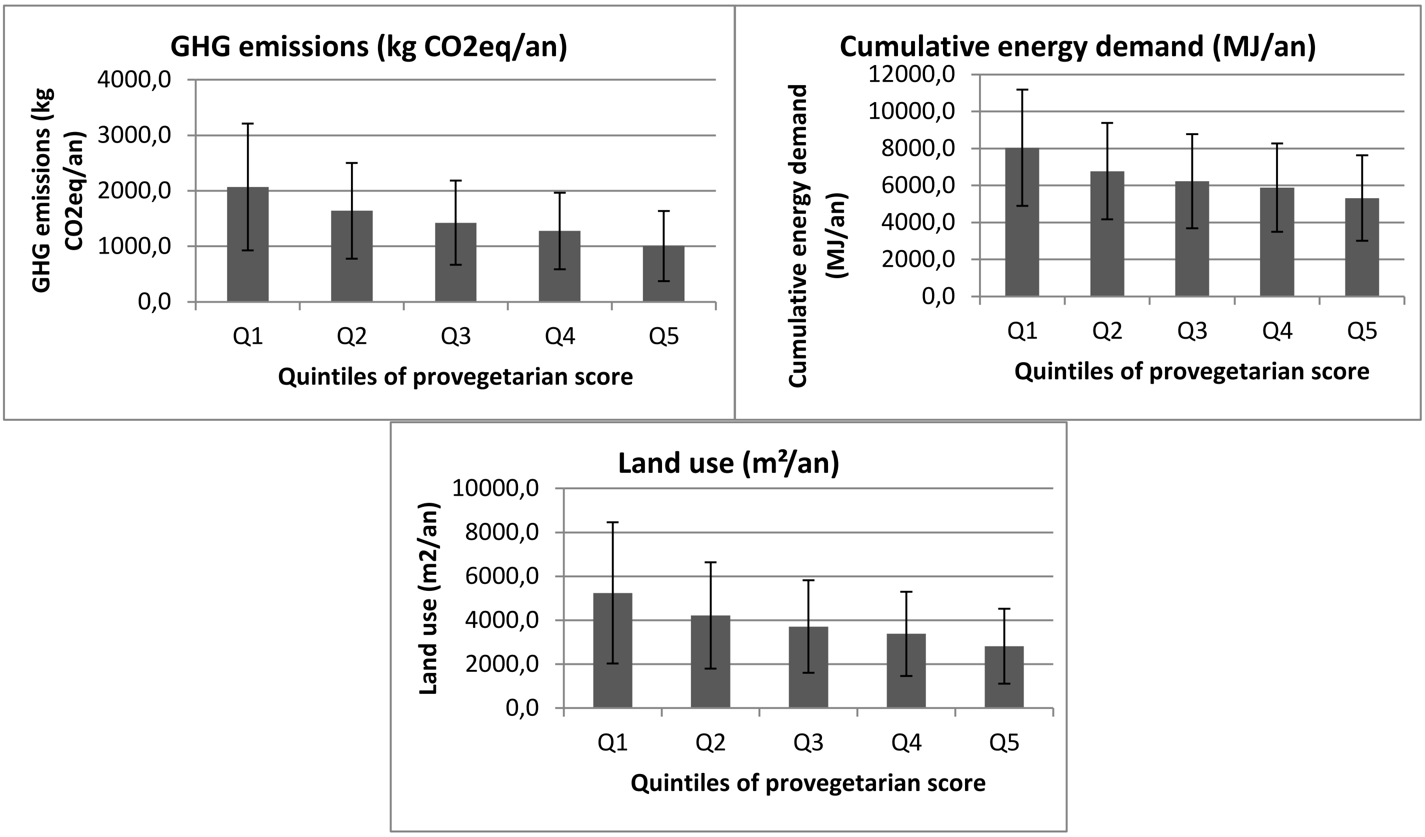

Means and SD are presented. P-trend<0.0001 for all indicators estimated using contrast linear test.
